# Correlates and consequences of atrial fibrillation in a prospective study of 25 000 participants in the China Kadoorie Biobank

**DOI:** 10.1093/ehjopen/oeae021

**Published:** 2024-03-19

**Authors:** Iain Turnbull, Christian Fielder Camm, Jim Halsey, Huaidong Du, Derrick A Bennett, Yiping Chen, Canqing Yu, Dianyianji Sun, Xiaohong Liu, Liming Li, Zhengming Chen, Robert Clarke, China Kadoorie Biobank Study Group, Junshi Chen, Zhengming Chen, Robert Clarke, Rory Collins, Liming Li, Jun Lv, Richard Peto, Robin Walters, Daniel Avery, Derrick Bennett, Ruth Boxall, Ka Hung Chan, Yiping Chen, Zhengming Chen, Charlotte Clarke, Johnathan Clarke, Robert Clarke, Huaidong Du, Geoffrey Ma, Ahmed Edris Mohamed, Hannah Fry, Simon Gilbert, Pek Kei Im, Andri Iona, Maria Kakkoura, Christiana Kartsonaki, Hubert Lam, Kuang Lin, James Liu, Mohsen Mazidi, Iona Millwood, Sam Morris, Qunhua Nie, Alfred Pozarickij, Maryam Rahmati, Paul Ryder, Dan Schmidt, Becky Stevens, Iain Turnbull, Robin Walters, Baihan Wang, Lin Wang, Neil Wright, Ling Yang, Xiaoming Yang, Pang Yao, Xiao Han, Can Hou, Qingmei Xia, Chao Liu, Jun Lv, Pei Pei, Dianjianyi Sun, Canqing Yu, Lang Pan, Naying Chen, Duo Liu, Zhenzhu Tang, Ningyu Chen, Qilian Jiang, Jian Lan, Mingqiang Li, Yun Liu, Fanwen Meng, Jinhuai Meng, Rong Pan, Yulu Qin, Ping Wang, Sisi Wang, Liuping Wei, Liyuan Zhou, Caixia Dong, Pengfei Ge, Xiaolan Ren, Zhongxiao Li, Enke Mao, Tao Wang, Hui Zhang, Xi Zhang, Jinyan Chen, Ximin Hu, Xiaohuan Wang, Zhendong Guo, Huimei Li, Yilei Li, Min Weng, Shukuan Wu, Shichun Yan, Mingyuan Zou, Xue Zhou, Ziyan Guo, Quan Kang, Yanjie Li, Bo Yu, Qinai Xu, Liang Chang, Lei Fan, Shixian Feng, Ding Zhang, Gang Zhou, Yulian Gao, Tianyou He, Pan He, Chen Hu, Huarong Sun, Xukui Zhang, Biyun Chen, Zhongxi Fu, Yuelong Huang, Huilin Liu, Qiaohua Xu, Li Yin, Huajun Long, Xin Xu, Hao Zhang, Libo Zhang, Jian Su, Ran Tao, Ming Wu, Jie Yang, Jinyi Zhou, Yonglin Zhou, Yihe Hu, Yujie Hua, Jianrong Jin, Fang Liu, Jingchao Liu, Yan Lu, Liangcai Ma, Aiyu Tang, Jun Zhang, Liang Cheng, Ranran Du, Ruqin Gao, Feifei Li, Shanpeng Li, Yongmei Liu, Feng Ning, Zengchang Pang, Xiaohui Sun, Xiaocao Tian, Shaojie Wang, Yaoming Zhai, Hua Zhang, Wei Hou, Silu Lv, Junzheng Wang, Xiaofang Chen, Xianping Wu, Ningmei Zhang, Weiwei Zhou, Xiaofang Chen, Jianguo Li, Jiaqiu Liu, Guojin Luo, Qiang Sun, Xunfu Zhong, Weiwei Gong, Ruying Hu, Hao Wang, Meng Wang, Min Yu, Lingli Chen, Qijun Gu, Dongxia Pan, Chunmei Wang, Kaixu Xie, Xiaoyi Zhang

**Affiliations:** Clinical Trial Service Unit, Nuffield Department of Population Health, University of Oxford, Old Road Campus, Headington, Oxford, OX3 7LF, UK; Clinical Trial Service Unit, Nuffield Department of Population Health, University of Oxford, Old Road Campus, Headington, Oxford, OX3 7LF, UK; Clinical Trial Service Unit, Nuffield Department of Population Health, University of Oxford, Old Road Campus, Headington, Oxford, OX3 7LF, UK; Clinical Trial Service Unit, Nuffield Department of Population Health, University of Oxford, Old Road Campus, Headington, Oxford, OX3 7LF, UK; Clinical Trial Service Unit, Nuffield Department of Population Health, University of Oxford, Old Road Campus, Headington, Oxford, OX3 7LF, UK; Clinical Trial Service Unit, Nuffield Department of Population Health, University of Oxford, Old Road Campus, Headington, Oxford, OX3 7LF, UK; Department of Epidemiology and Biostatistics, Peking University, Beijing, China; Department of Epidemiology and Biostatistics, Peking University Center for Public Health and Epidemic Preparedness and Response, Beijing, China; Department of Epidemiology and Biostatistics, Peking University, Beijing, China; Department of Epidemiology and Biostatistics, Peking University Center for Public Health and Epidemic Preparedness and Response, Beijing, China; Medical Records Archive, Pengzhou Traditional Medicine Hospital, Penzhou, China; Department of Epidemiology and Biostatistics, Peking University, Beijing, China; Department of Epidemiology and Biostatistics, Peking University Center for Public Health and Epidemic Preparedness and Response, Beijing, China; Key Laboratory of Epidemiology of Major Diseases, Peking University, Beijing, China; Clinical Trial Service Unit, Nuffield Department of Population Health, University of Oxford, Old Road Campus, Headington, Oxford, OX3 7LF, UK; Clinical Trial Service Unit, Nuffield Department of Population Health, University of Oxford, Old Road Campus, Headington, Oxford, OX3 7LF, UK

**Keywords:** Atrial fibrillation, Left ventricular hypertrophy, Ischaemia, Stroke and ischaemic heart disease

## Abstract

**Aims:**

The prevalence of atrial fibrillation (AF) is positively correlated with prior cardiovascular diseases (CVD) and CVD risk factors but is lower in Chinese than Europeans despite their higher burden of CVD. We examined the prevalence and prognosis of AF and other electrocardiogram (ECG) abnormalities in the China Kadoorie Biobank.

**Methods and results:**

A random sample of 25 239 adults (mean age 59.5 years, 62% women) had a 12-lead ECG recorded and interpreted using a Mortara VERITAS™ algorithm in 2013–14. Participants were followed up for 5 years for incident stroke, ischaemic heart disease, heart failure (HF), and all CVD, overall and by CHA_2_DS_2_-VASc scores, age, sex, and area. Overall, 1.2% had AF, 13.6% had left ventricular hypertrophy (LVH), and 28.1% had ischaemia (two-thirds of AF cases also had ischaemia or LVH). The prevalence of AF increased with age, prior CVD, and levels of CHA₂DS₂-VASc scores (0.5%, 1.3%, 2.1%, 2.9%, and 4.4% for scores <2, 2, 3, 4, and ≥5, respectively). Atrial fibrillation was associated with two-fold higher hazard ratios (HR) for CVD (2.15; 95% CI, 1.71–2.69) and stroke (1.88; 1.44–2.47) and a four-fold higher HR for HF (3.79; 2.21–6.49). The 5-year cumulative incidence of CVD was comparable for AF, prior CVD, and CHA₂DS₂-VASc scores ≥ 2 (36.7% vs. 36.2% vs. 37.7%, respectively) but was two-fold greater than for ischaemia (19.4%), LVH (18.0%), or normal ECG (14.1%), respectively.

**Conclusion:**

The findings highlight the importance of screening for AF together with estimation of CHA₂DS₂-VASc scores for prevention of CVD in Chinese adults.

## Graphical Abstract

**Graphical Abstract:**
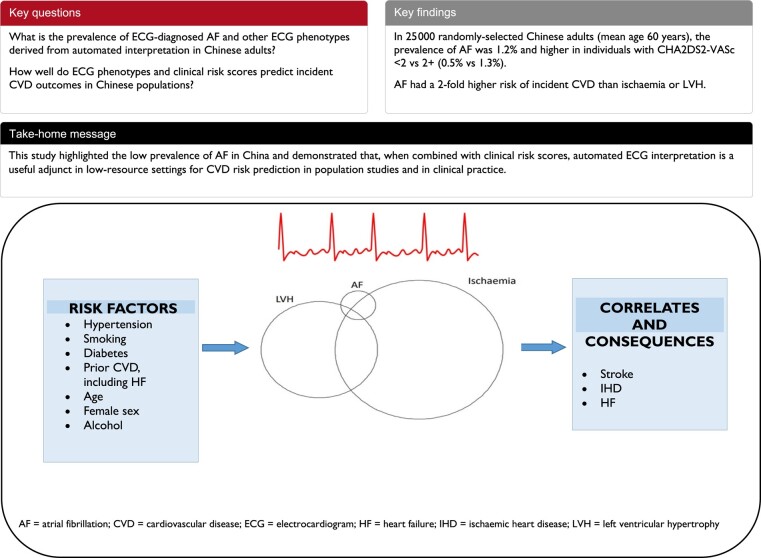
Correlates and consequences of atrial fibrillation.

## Introduction

Ischaemic heart disease (IHD) and stroke are the leading causes of death and disability in China^1^ where the mortality rates are higher than in other Asian countries.^2^ Moreover, years of life lost (YLLs) due to IHD and stroke in China greatly exceed those in Western countries.^3^ While highly correlated with the burden of prior cardiovascular disease (CVD) or levels of CVD risk factors,^4^ the prevalence of atrial fibrillation (AF) is lower in Chinese than in Western populations.^5,6^ The reasons for the discrepant prevalence estimates of AF between Chinese and European adults are not fully understood but may reflect differences in levels of CVD risk factors [height, body mass index (BMI), low-density lipoprotein cholesterol (LDL-C), genetic factors, alcohol, or smoking^7–10^] or low awareness and related detection errors.^11^ These differences are important as they not only influence risk of subsequent CVD outcomes but also increase the proportion of ischaemic stroke types due to cardioembolic (CE) causes, whose incidence differs markedly between Chinese and European ancestry populations.^12–14^

Previous population studies in China reported a substantial variation in the prevalence, incidence, and mortality of CVD between geographical areas within China.^15,16^ Likewise, prevalence of AF also varies between areas within China.^11,17^ Since AF is frequently asymptomatic, discrepancies in the reported prevalence of AF between studies may reflect differences in diagnostic practice and underestimate disease associations in prospective studies.^18^ Previous retrospective studies have estimated a high prevalence of undiagnosed AF, which is also associated with a high risk of stroke.^19^ In contrast, population studies involving universal ECG screening and automated interpretation of 12-lead ECGs should provide more reliable estimates of AF prevalence^20^ than those detected in routine clinical practice.^21,22^ However, the public health relevance of targeted screening to detect AF in low-resource settings such as China is uncertain.^23^

Several risk scores have been introduced to assess risk of CVD outcomes in individuals with and without AF. The CHA₂DS₂-VASc score^24,25^ was developed for use in Western populations to be more inclusive of risk factors for stroke, including non-stroke vascular disease, age, and sex, and is believed to provide more accurate risk stratification for stroke, in low-risk patients^26^ than its predecessor, CHADS_2_.^27^ The CHA₂DS₂-VASc score has been previously validated in a large population of Chinese adults with AF.^28^

Little is known about the effectiveness of population screening and automated reporting of 12-lead ECGs with or without concomitant CHA_2_DS_2_-VASc scores for prediction of major CVD outcomes in China or other low- and middle-income populations (LMIC). The aims of the present report were to (i) determine the prevalence of AF and other ECG abnormalities in a random sample of 25 239 participants in the China Kadoorie Biobank (CKB), overall and by age, sex, area, prior CVD, and levels of CVD risk factors including CHA_2_DS_2_-VASc scores, and (ii) compare the prognosis of AF and other ECG abnormalities by CHA_2_DS_2_-VASc scores with subsequent CVD outcomes.

## Methods

### Study population

Details of the study design, methods, and participants of the CKB have been described elsewhere.^29^ Briefly, 512 726 men and women, aged 30–79 years, were recruited from 10 diverse (5 urban and 5 rural) areas in China. The baseline survey was conducted during 2004–08 by trained health workers in local study assessment centres. Information was collected using interview-administered questionnaires including data collection on demographic and socioeconomic status, medical history, and lifestyle factors. Measurements of blood pressure, height, weight, and lung function were recorded, and a blood sample was collected for long-term storage. During 2013–14, a second resurvey was conducted, using administrative units (i.e. rural village or urban residential committee) as primary sampling units, on ∼5% of randomly selected surviving participants. The resurvey collected identical data to those collected at baseline, but, in addition, all participants had a 12-lead ECG recorded. A modified form of CHA_₂_DS_₂_-VASc score^24^ was estimated for each participant and used to stratify participants by prior risk at the second resurvey (see Supplementary material online, *Methods* for additional details: Supplementary material online, *Methods S1A* and *B*). Ethics approval was obtained from all relevant local, national, and international ethics committees prior to enrolment in the study. All participants provided written informed consent to participate in the study and permission for study investigators to access their medical records for research purposes.

### Electrocardiogram assessment

At the second resurvey, ECG measurements were performed using a Mortara ELI^TM^ 250c resting ECG machine (Welch Allyn, Inc. Skaneateles Falls, NY, USA). Standard operating procedures were used to train staff at all regional centres (see Supplementary material online, *Methods*). Using the Mortara VERITAS™ ECG algorithm, up to 255 individual text strings were generated automatically from a single ECG recording. Categories of ECG phenotypes were developed from automated ECG analysis by a cardiologist and a physician with relevant training in cardiology. The abnormalities were classified as: arrhythmias [including AF/atrial flutter (referred to as AF), bradyarrhythmias and heart block, or other arrhythmias/tachycardias], left ventricular hypertrophy (LVH), or myocardial ischaemia (including definite or probable/possible ischaemia). Additional descriptions of all ECG phenotypes are provided in the Supplementary material online, *Methods* (see Supplementary material online, *Methods S2*).

All ECG recordings in one area (Suzhou, *n* = 2860 participants; 11% of total) had a single limb lead misplacement error in which the leads for the left arm and left leg were reversed, which was likely to alter the amplitude of the S-waves in aVL. Consequently, text strings for LVH and non-specific ECG phenotypes may have been incorrectly assigned by automated ECG interpretation software in the Suzhou area. While text strings for AF and other rhythm disorders should have remained unaffected, it is possible that the extent (or severity) of algorithmically assigned ischaemia (definite/probable/possible) may have been altered by this limb lead reversal error. New text strings could not be generated in this regional subset as the Mortara VERITAS™ algorithm is proprietary.

### Follow-up and outcome measures

Participants were followed up by electronic linkage, using a unique personal identification number, to death and disease-specific registers and to nationwide health insurance system (which had >97% coverage at enrolment) for all hospitalization episodes, supplemented by annual active follow-up to minimize any loss to follow-up.^29^ All fatal and non-fatal disease outcomes were coded by trained clinical staff using the International Classification of Diseases 10th Revision (ICD-10). The incident CVD outcomes recorded after resurvey in the present study included stroke (I60–I61, I63–I64), IHD (I20–I25), HF (I50), and CVD (I00–I99). Participants contributed person-years from time of resurvey until the date of their outcome of interest or a censoring date of 31 December 2018.

### Statistical analysis

The present analyses included all 25 239 participants with ECG data at resurvey. Mean values and proportions of characteristics at resurvey were estimated by sex, modified CHA₂DS₂-VASc scores, and prior self-reported CVD, respectively. The proportions of ECG phenotypes were also evaluated by age and modified CHA₂DS₂-VASc scores and separately by sex and area.

Cox proportional hazards models were used to estimate hazard ratios (HR) for associations of ECG phenotypes with incident CVD, stratified by age at risk, sex, and area and adjusted for self-reported smoking and alcohol drinking. Analyses of associations of modified CHA₂DS₂-VASc scores (as categorical variables) with disease outcomes used group-specific variances to estimate the 95% confidence intervals (CI) for all categories enabling comparisons between any two categories and not only with the reference group.^30^

Kaplan–Meier curves for fatal and non-fatal incident CVD outcomes were plotted separately by age groups and weighted by the number of participants in each group for each ECG phenotype. Since age was included in the construction of CHA₂DS₂-VASc scores, no additional adjustment for age was used when estimating cumulative incidence of CVD outcomes. In addition, all analyses of LVH, other non-specific ECG changes, and all ischaemia were repeated following the exclusion of participants from the Suzhou area with limb lead misplacement error. All analyses were conducted using R version 4.3 or SAS version 9.4 and involved CKB data from release 18.0. Scaled Venn diagrams were generated using the eulerr R library.

## Results

Baseline characteristics of study participants at resurvey are shown by the presence or absence of prior CVD or CHA₂DS₂-VASc scores in *Table 1*. The mean [standard deviation (SD)] age was 59.5 (10.2) years, 61.7% were women, and 56.7% lived in rural areas. Two-thirds of men were ever-regular smokers or ever-regular drinkers, but rates of smoking and drinking were much lower in women (2.9% and 18.6%, respectively). Analyses stratified by modified CHA₂DS₂-VASc scores indicated that 12 509 (49.6%) participants had scores ≥ 2 and, compared with those with scores < 2, were older [mean (SD) age 65.1 (9.6) vs. 54.0 (7.4) years] and predominantly women (75.2% vs. 48.4%), and had higher mean levels of systolic blood pressure (SBP), BMI, and random plasma glucose (RPG) (*Table 1*).

**Table 1:** Selected characteristics of 25 000 individuals with electrocardiogram records

	Has prior CVD		CHA_2_DS_2_-VASc		All
	No	Yes	<2	2+	
**No. of participants**	**(*n* = 23 056)**	**(*n* = 2183)**	**(*n* = 12 730)**	**(*n* = 12 509)**	**(*n* = 25 239)**
**Age at ECG, mean(SD), years**	58.9 (10.1)	65.7 (9.0)	54.0 (7.4)	65.1 (9.6)	59.5 (10.2)
**Sex**					
Female, %	61.9	59.9	48.4	75.2	61.7
**Area**					
Rural, %	57.9	44.0	60.1	53.2	56.7
**Ever-regular smoker, %**					
Men	67.2	65.9	68.6	63.8	67.1
Women	2.9	2.7	2.6	3.1	2.9
**Ever-regular drinker, %**					
Men	65.9	60.2	69.2	57.5	65.4
Women	18.7	17.3	23.3	15.6	18.6
**Clinical measures, mean (SD)**					
SBP, mmHg	136 (20.5)	144 (21.5)	126 (15.6)	147 (19.9)	137 (20.7)
BMI, kg/m^2^	24.1 (3.5)	25.3 (3.7)	23.8 (3.2)	24.6 (3.7)	24.2 (3.5)
Waist/hip ratio	0.9 (0.1)	0.9 (0.1)	0.9 (0.1)	0.9 (0.1)	0.9 (0.1)
RPG, mmol/L	5.9 (2.2)	6.4 (2.5)	5.5 (1.4)	6.4 (2.7)	6.0 (2.2)
**Medical history** ^a^					
Heart failure, %	0.1	0.7	0.0	0.3	0.2
Hypertension, %	47.9	75.6	18.1	83.0	50.3
Diabetes, %	8.9	18.1	1.6	18.0	9.7
Stroke/TIA, %	0.0	39.2	0.0	6.8	3.4
Non-stroke CVD, %	0.0	60.8	0.5	10.1	5.3

ECG, electrocardiogram; SD, standard deviation; SBP, systolic blood pressure; BMI, body mass index; RPG, random plasma glucose; CVD, cardiovascular disease; DBP, diastolic blood pressure.

^a^Prior diseases all self-reported except heart failure, which is reported from hospitalized episodes; hypertension, which includes self-reported disease and those with SBP ≥ 140 mmHg and DPB ≥ 90 mmHg at resurvey; and diabetes, which includes self-reported disease and those with raised RPG at resurvey.

Conversely, about 8.6% (*n* = 2183) of participants had a prior history of CVD, but these had a similar age to those with modified CHA₂DS₂-VASc ≥ 2 [mean age 65.7 (9.0)] (*Table 1*). Participants with modified CHA₂DS₂-VASc < 2 were younger than those without prior CVD and had lower levels of SBP, BMI, and RPG. Importantly, the prevalence of prior hypertension was substantially lower in participants with modified CHA₂DS₂-VASc < 2 than in those without prior CVD and likewise for diabetes (*Table 1*). Conversely, 41.2% (*n* = 10 386) individuals with a modified CHA₂DS₂-VASc ≥ 2 had prevalent hypertension (see Supplementary material online, *Table S1*). The prevalence of prior stroke/TIA and non-stroke CVD was about six-fold greater in those with prior CVD than those with modified CHA₂DS₂-VASc ≥ 2 (39.2% vs. 6.8% and 60.8% vs. 10.1%, respectively).

**Figure 1:**
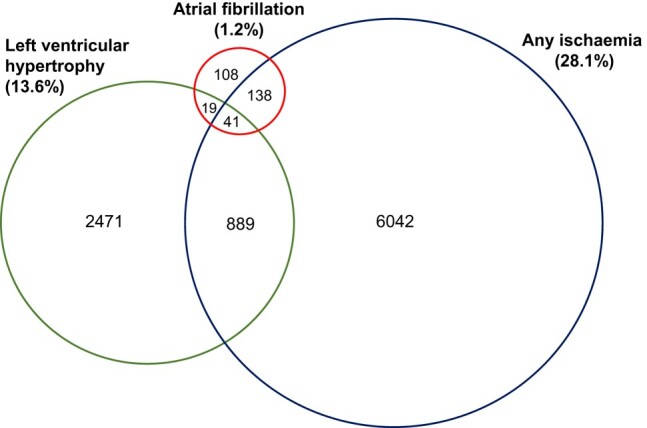
Prevalence (%) and overlapping distributions of numbers of cases with atrial fibrillation, left ventricular hypertrophy, and any ischaemia.

*Table 2* shows the prevalence of ECG phenotypes by age or CHA₂DS₂-VASc scores. Overall, 1.2% (95% CI 1.07–1.33; *n* = 306 cases) had AF on automated reporting (*Table 2*). *Figure 1* shows the overlapping distributions of AF, ischaemia, and LVH. A total of 58.5% and 19.6% of participants with AF had ECG evidence of ischaemia and LVH, respectively, while 13.4% had features of both. The prevalence of AF increased with age at resurvey (0.4%, 0.9%, and 3.9% at <55, 55–69, and ≥70 years, respectively) and CHA₂DS₂-VASc scores (0.5%, 1.3%, 2.1%, 2.9%, and 4.4%; score <2, 2, 3, 4, and ≥5, respectively) in both men and women (see Supplementary material online, *Table S2A* and *B*). Among all 306 participants with an automated diagnosis of the AF, 44 (14.4%) had evidence of prior incident hospitalization with AF. Among individuals with AF, 79.0% (*n* = 242) had CHA₂DS₂-VASc ≥ 2 and 50.0% (*n* = 153) had a score ≥ 3, respectively. The prevalence of ECG-detected AF varied three-fold by area [Qingdao (2.5%) vs. Henan (0.9%): Supplementary material online, *Table S3*] and was more frequent in urban than in rural areas. Likewise, AF was more common in men than women (1.6% vs. 1.0%, respectively).

**Table 2:** Prevalence of electrocardiogram phenotypes by age and CHA_2_DS_2_-VASc score

ECG phenotypes	Age (years) at ECG measurement			CHA_2_DS_2_-VASc					All
	<55	55–69	70+	<2	2	3	4	5+	
**Number of participants**	9149	11 729	4361	12 730	6671	3538	1668	632	25 239
**Arrhythmia/pro-arrhythmia, *N* (%)**									
Atrial fibrillation/flutter	34 (0.4)	100 (0.9)	172 (3.9)	64 (0.5)	89 (1.3)	76 (2.1)	49 (2.9)	28 (4.4)	306 (1.2)
Pro-AF condition^a^	98 (1.1)	238 (2.0)	218 (5.0)	157 (1.2)	169 (2.5)	118 (3.3)	76 (4.6)	34 (5.4)	554 (2.2)
Other arrhythmia/tachycardia	622 (6.8)	1004 (8.6)	662 (15.2)	944 (7.4)	599 (9.0)	432 (12.2)	222 (13.3)	91 (14.4)	2288 (9.1)
Bradycardia/heart block	442 (4.8)	762 (6.5)	389 (8.9)	802 (6.3)	396 (5.9)	239 (6.8)	117 (7.0)	39 (6.2)	1593 (6.3)
**Ischaemia, *N* (%)**									
Possible/probable	1853 (20.3)	3216 (27.4)	1417 (32.5)	2353 (18.5)	2012 (30.2)	1223 (34.6)	642 (38.5)	256 (40.5)	6486 (25.7)
Definite	181 (2.0)	286 (2.4)	157 (3.6)	279 (2.2)	161 (2.4)	108 (3.1)	51 (3.1)	25 (4.0)	624 (2.5)
**Left ventricular hypertrophy, *N* (%)**	906 (9.9)	1731 (14.8)	783 (18.0)	1535 (12.1)	972 (14.6)	558 (15.8)	260 (15.6)	95 (15.0)	3420 (13.6)
**Non-specific ECG changes, *N* (%)**	1106 (12.1)	1788 (15.2)	1046 (24.0)	1883 (14.8)	952 (14.3)	661 (18.7)	330 (19.8)	114 (18.0)	3940 (15.6)
**Normal variant, *N* (%)**	4837 (52.9)	5071 (43.2)	1284 (29.4)	6506 (51.1)	2779 (41.7)	1218 (34.4)	506 (30.3)	183 (29.0)	11 192 (44.3)

Percentages are calculated separately within columns.

ECG, electrocardiogram; AF, atrial fibrillation.

^a^Pro-AF condition: phenotype comprising ECG features that may increase the risk of development of atrial fibrillation.

A total of 7110 (28.2%) participants had ischaemia [624 (8.8%) of which had definite ischaemia], and 3420 (13.6%) had LVH (*Table 2*). Both ischaemia and LVH phenotypes were more common in older individuals (*Table 2*) in both men and women (see Supplementary material online, *Table S2A* and *B*). In contrast, a total of 11 192 (44.3%) participants had normal ECG recordings (*Table 2*), and the prevalence of normal ECG declined with increasing age and CHA₂DS₂-VASc scores.

While the prevalence of the possible/probable ischaemic phenotype was higher in women than in men (28.9% vs. 20.5%), definite ischaemia was more commone in men (3.3% vs. 2.0%). Left ventricular hypertrophy was also more common in men than in women (19.0% vs. 10.2%) (see Supplementary material online, *Table S2A* and *B*). Analyses by area indicated that regional differences for ischaemia [Qingdao (34.8%) vs. Sichuan (22.2%)] differed from those for LVH [Qingdao (2.5%) vs. Sichuan (19.8%): Supplementary material online, *Table S3*]. Sensitivity analyses excluding participants from Suzhou (with limb lead error) did not materially alter the prevalence of ECG phenotypes (see Supplementary material online, *Table S4*).

**Figure 2:**
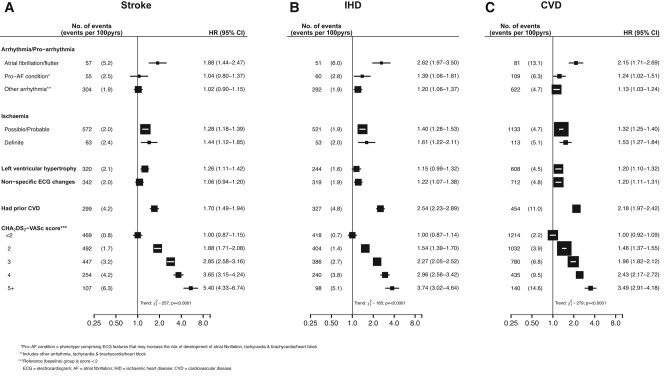
Associations of selected electrocardiogram phenotypes, history of prior cardiovascular disease, and CHA_2_DS_2_-VASc scores with first incident cases of (*A*) stroke, (*B*) ischaemic heart disease, and (*C*) any cardiovascular disease outcome. Values shown are the hazard ratios (HR) and 95% confidence intervals (CI).

*Figure 2* shows the associations of ECG phenotypes with incident stroke, IHD, and all CVD outcomes. Atrial fibrillation was associated with a two-fold higher risk of incident CVD of all types (HR 2.15; 95% CI 1.71–2.69) (*Figure 2*) and a four-fold higher risk of incident HF (HR 3.79; 2.21–6.49) (see Supplementary material online, *Figure S1*). Atrial fibrillation was associated with a comparable HR of stroke as a modified CHA₂DS₂-VASc ≥ 2 or history of prior CVD (*Figure 2*). The strength of associations of modified CHA₂DS₂-VASc scores with each CVD outcome increased linearly with higher scores (*Figure 2*: *P* for trend ≤0.0001 for all outcomes) but was most strongly associated with HF (see Supplementary material online, *Figure S1*: *P* for trend ≤0.0001).

The presence of ischaemia was associated with higher risks of IHD (*Figure 2*) and HF (see Supplementary material online, *Figure S1*). In contrast, LVH was more strongly associated with stroke (*Figure 2*). Sensitivity analyses demonstrated that the associations of LVH, non-specific ECG, and ischaemic phenotypes with all CVD outcomes were unaltered by exclusion of participants from Suzhou (see Supplementary material online, *Figure S2*).

**Figure 3:**
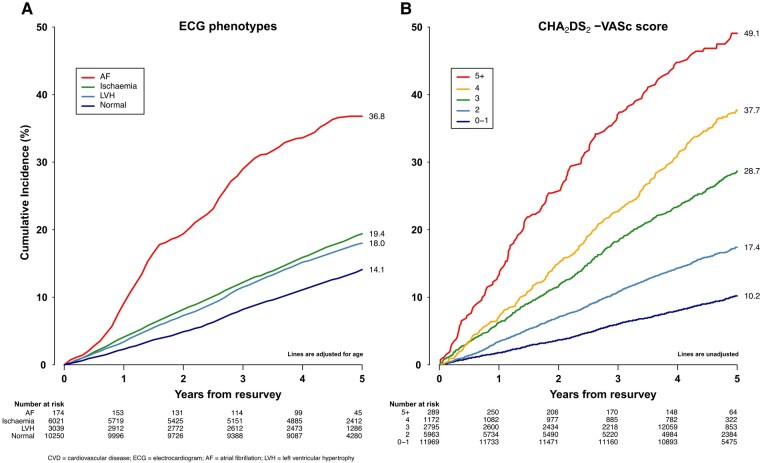
Cumulative incidence of first incident cardiovascular disease outcomes by (*A*) electrocardiogram phenotypes and (*B*) CHA_2_DS_2_-VASc scores. The electrocardiogram phenotypes evaluated were atrial fibrillation, ischaemia, left ventricular hypertrophy, and normal electrocardiogram tracing.

*Figure 3* shows the cumulative incidence of any CVD outcome by major ECG phenotypes compared with CHA₂DS₂-VASc scores. After adjustment for age, the 5-year cumulative incidence of CVD was 36.8% in individuals with AF, 19.4% in those with ischaemia, 18.0% in those with the LVH, and 14.1% in those with a normal ECG (*Figure 3*). Individuals living in rural areas had a higher cumulative incidence of CVD after 5 years of follow-up than those from urban areas (37.4% vs. 32.2%, respectively) (see Supplementary material online, *Figure S3*).

The cumulative incidence of CVD associated with all ECG phenotypes was comparable in men and women (see Supplementary material online, *Figure S4*). Cumulative incidence of CVD also increased for each increment of modified CHA₂DS₂-VASc score, with nearly three-fold differences between participants with scores of ≥5 vs. ≥2 (49.1% vs. 17.4%, respectively) (*Figure 3*). History of prior CVD was associated with two-fold differences in cumulative incidence of CVD (36.2% vs. 15.3%, respectively) (see Supplementary material online, *Figure S5*). Men with modified CHA₂DS₂-VASc ≥ 2 had a higher cumulative incidence of CVD than women with scores ≥ 3 (33.6% vs. 29.8%) (see Supplementary material online, *Figure S4*). Sensitivity analyses after exclusion of individuals from Suzhou area did not alter the cumulative incidence of CVD for ECG phenotypes or modified CHA₂DS₂-VASc scores (see Supplementary material online, *Figure S6*).

## Discussion

This population-based study of 25 239 Chinese adults with automated interpretation of ECG recordings demonstrated that the prevalence of AF was 1.2%, consistent with previous reports from China^11,17^ and other East Asian studies,^31,32^ but was much lower than estimates in Western populations.^33,34^ In Chinese adults, the prevalence of AF was more common in men, increased with age and higher CHA₂DS₂-VASc scores, and was higher in urban than rural areas. Importantly, two-thirds of AF cases also had some evidence of ischaemia or LVH, perhaps reflecting shared aetiology.

Likewise, the prevalence of ECG-detected LVH and ischaemia also increased by age and higher CHA₂DS₂-VASc scores. Over three-quarters of study individuals with a CHA₂DS₂-VASc score of ≥2 had hypertension at time of resurvey. A previous analyses of 0.5 M adults in the overall CKB study population demonstrated one-third had hypertension at baseline, with much lower levels of diagnosis, treatment, and control than in Western populations.^35^ About two-thirds of men in the present resurvey population were both regular smokers and regular drinkers, and about one-sixth of resurvey participants with prior CVD or a CHA₂DS₂-VASc score of ≥2 had self-reported diabetes or a raised random plasma glucose. Likewise for AF, the prevalence and patterns of alcohol consumption in Chinese adults differed considerably by sex and geographical area.^36^ Conversely, while overweight and obesity in China have increased substantially in recent decades, the mean levels of BMI in China were lower than in Western populations.^37^ The associations of hypertension, heavy alcohol intake, tobacco use, obesity, and diabetes with incident AF and other CVD types are well-established^38^ and several pathophysiological mechanisms, such as atrial stretch, ischaemia, and structural remodelling,^39,40^ may contribute to the aetiology of AF.

Among all 306 participants with an automated diagnosis of AF, <15% had evidence of prior hospitalization episodes with AF, but nearly a half to three-quarters had a modified CHA₂DS₂-VASc score of ≤2 and ≤3, respectively. Compared with symptomatic AF, asymptomatic AF has been independently associated with higher risks of stroke and death,^41–44^ with one US study showing significantly lower CHA₂DS₂-VASc scores in cases with symptoms (e.g. palpitations, fatigue, dyspnoea, dizziness) than in asymptomatic presentations.^44^ Atrial fibrillation detected at or after admission to hospital for treatment of stroke, known as ‘silent’ AF,^45^ may be detected by serial ECGs, Holter monitoring, continuous telemetry, or continuous ECG monitoring during hospitalization and using implantable loop recorders and outpatient telemetry after discharge. In a meta-analysis of over 10 000 cases with Ischaemic Stroke (IS), a diagnosis of new or silent AF was detected in 23.7%.^46^ Although lower rates of AF in China may account for the lower proportions of IS cases with CE aetiology observed in Chinese adults,^13,14,47^ the true frequency of AF-associated stroke in China may have been underestimated by suboptimal detection by 12-lead ECG rather than detection by prolonged ECG monitoring in clinical practice.^48^

In 2018, the US Preventive Services Task Force (USPSTF) advocated against population screening using resting or exercise ECG to screen for CVD in asymptomatic adults in low-risk settings.^49^ For adults at intermediate or high risk, there was insufficient evidence to assess the impact of ECG screening for CVD risk assessment and primary prevention of CVD outcomes. Likewise, the UK National Screening Committee concluded that using 12-lead ECG to detect AF in asymptomatic individuals would not fulfil the optimum criteria for a screening test,^50^ citing uncertainty around benefits of treating screen-detected arrhythmia. In contrast, in 2020, the revised European guidelines advocated that systematic screening of all older adults for AF should be considered.^51^

Randomized controlled trials (RCTs) conducted in the USA^52^ and Netherlands^53^ compared opportunistic screening using single-lead ECG with usual care, both in patients aged ≥65 years without a previous diagnosis of AF, and found no difference in rates of detection of AF between intervention and control groups. However, in 2017, the UK-based REHEARSE-AF Study, which compared ambulatory single-lead ECG with routine care in AF-free patients aged ≥65 years, demonstrated a nearly four-fold higher incidence of AF but showed no differences in stroke and TIA during the 12-month follow-up period.^54^ Moreover, the STROKESTOP Study, conducted in Sweden during 2012–14 among older individuals (aged 75–76 years) allocated to intermittent single-lead ECG or usual care,^55^ showed a similar increase in detection of AF cases, and in an allied RCT examining CVD outcomes,^56^ a small net clinical benefit was demonstrated over standard care, with fewer CVD outcomes. Screening also increased the detection of AF by one-third, and anticoagulant therapy was initiated in >90% of those with newly identified cases, while in another trial in the USA, involving randomization to a wearable continuous ECG monitoring patch, similar treatment initiation rates were observed.^57^ While systematic population-based screening for AF is limited by low detection rates, uncertain cost-effectiveness, and doubts over feasibility in clinical practice, selective screening of high-risk individuals for CVD significantly increases detection rates and reduces the number needed to screen.^58^

Recent guidelines recommended that, unless otherwise contraindicated, anticoagulation should be commenced in people with a CHA₂DS₂-VASc score of ≥2 if male and of ≥3 if female.^51^ In CKB, the risks of CVD were over two-fold greater for individuals with prior self-reported CVD, consistent with those with modified CHA₂DS₂-VASc scores of ≥3–4. A history of prior CVD was more strongly associated with subsequent CVD than with incident stroke (HR 2.18 vs. 1.70, respectively). However, modified CHA₂DS₂-VASc scores of ≥2 or 3 were more strongly associated with stroke than with CVD (HR 1.88 or 2.85 vs. HR 1.46 or 1.96, respectively).

While phenotypes for ischaemia, LVH, and other arrhythmias were associated with moderately increased risks of incident CVD, AF was more strongly and positively associated with CVD outcomes, with HRs for stroke comparable with those for prior CVD or a modified CHA₂DS₂-VASc score of ≥2. During 5 years of follow-up, about one-third of participants with AF had incident CVD, which was two-fold greater than the cumulative incidence for other ECG phenotypes and comparable with incidence associated with modified CHA₂DS₂-VASc scores of ≥3 and 4. Individuals with a normal ECG had a lower incidence of CVD than those with ischaemia and LVH, consistent with previous community-based population studies using automatic reporting of ECGs.^59,60^

Automated interpretation of 12-lead ECGs has a higher specificity but equal sensitivity for AF diagnosis compared with interpretation by healthcare professionals.^20^ However, despite expansion of the primary care system within China,^61^ there have been no nationally representative appraisals of the quality of primary care in rural areas. In a 2020 cross-sectional study involving 144 village doctors across 5 provinces,^62^ clinicians were presented with mock patients alongside standardized clinical vignettes for unstable angina and their practice assessed against national guidelines. About 20% provided the correct diagnosis, and only 13% had on-site access to ECG machines. Moreover, findings from a large-scale, nationwide, community-based survey in China^11^ reported that over one-third of adults with AF were unaware of their diagnosis, particularly males and individuals living in rural areas, and only 6% were prescribed anticoagulant therapy.

With improved access to non-vitamin K antagonist oral anticoagulants (NOACs) in urban areas in China,^63^ inequalities in disease outcomes and use of treatments between rural and urban areas are likely to increase in future years. In CKB, participants from rural areas with AF identified on ECG had a higher cumulative incidence rate of CVD than those from urban areas (37.4% vs. 32.2%), which may reflect differences in access to treatments.

The chief strengths of the present study included the estimation of the prevalence and prognosis of AF and other ECG abnormalities using ECG screening with automated interpretation of 12-lead ECGs in addition to CHA_2_DS_2_-VASc scores in a random sample of 25 000 Chinese adults. However, the present study also had several important limitations. The strong association of AF with CVD outcomes compared with other ECG traits cannot fully exclude the confounding effects of age and prior history and treatment of CVD. Differences in the cumulative incidence of CVD between urban and rural areas may reflect differential access to anticoagulant therapy for AF, but the CKB study was unable to address this hypothesis as it did not collect data on symptoms or use of specific medications for AF. In addition, CKB is not representative of the overall Chinese adult population. The resurvey cohort was relatively young (mean age 59.5 years), and evaluation of the prevalence of AF and other phenotypes may have been underestimated. Finally, since ECG recordings were conducted at a single visit, the study is likely to have underestimated the prevalence of paroxysmal AF, which is associated with higher risks of thromboembolic outcomes.^44^

Nevertheless, this study highlighted paradoxical low prevalence of AF despite high CHA_2_DS_2_-VASc scores, with substantial overlap of AF with ischaemia and LVH suggesting shared aetiology, but further study is required to determine whether the low prevalence reflects lower levels of LDL-C or BMI or other unexplained factors. We also demonstrated the value of estimation of CHA_2_DS_2_-VASc scores to identify high-risk individuals to screen for AF and guide use of specific treatments for AF in low-resource populations for prevention of CVD outcomes.

## Supplementary Material

oeae021_Supplementary_Data

## Data Availability

The observational data that support the findings of this study are available to bona fide researchers on application under the CKB Open Access Data Policy (www.ckbiobank.org). Sharing of genotyping data is constrained by the Administrative Regulations on Human Genetic Resources of the People’s Republic of China. Access to these is available through collaboration with CKB researchers.

## Lead author biography

Iain Turnbull [BA(Hons) MB BChir MRCP(UK) MRCGP(2009) Clinical Research Fellow]studied medicine at University of Cambridge, graduating in 2003. He worked in general medicine in the NHS, becoming a Member of the Royal College of Physicians in 2007. Iain then completed training in general practice in 2009 and continues to practise in the South West of England. He joined Oxford Population Health in 2015 and works as a clinical research fellow for the CKB. His research involves the development of techniques for standardizing Chinese health insurance data and procedures for disease classification and adjudication.
