# Correction to: Correlates and consequences of atrial fibrillation in a prospective study of 25 000 participants in the China Kadoorie Biobank

**DOI:** 10.1093/ehjopen/oeae067

**Published:** 2024-08-21

This is a correction to: Iain Turnbull, Christian Fielder Camm, Jim Halsey, Huaidong Du, Derrick A Bennett, Yiping Chen, Canqing Yu, Dianyianji Sun, Xiaohong Liu, Liming Li, Zhengming Chen, Robert Clarke, China Kadoorie Biobank Study Group, Correlates and consequences of atrial fibrillation in a prospective study of 25 000 participants in the China Kadoorie Biobank, *European Heart Journal Open*, Volume 4, Issue 2, March 2024, oeae021, https://doi.org/10.1093/ehjopen/oeae021

The heading for the third and fourth columns of Table 1 has been corrected from ‘Left ventricular hypertrophy (Mortara)’ to read CHA2DS2-VASc. Subheadings of those columns have been corrected from ‘No’ to read ‘<2’ and from ‘Yes’ to read ‘2+’.

