# Supplementary material for: Correlates and consequences of atrial fibrillation in a prospective study of 25 000 participants in the China Kadoorie Biobank
Source: Eur Heart J Open. 2024 Mar 19;4(2):oeae021. doi: 10.1093/ehjopen/oeae021 (PMC10989653; doi:10.1093/ehjopen/oeae021)
Supplement: oeae021_Supplementary_Data [file oeae021_supplementary_data.zip › SOP-for-ECG recording in CKB.pdf]

中国慢性病前瞻性研究(CKB)

第二次复查

心电图检查

【标准操作手册】

2013 年 9 月

项目负责单位：中国医学科学院,英国牛津大学

目录

一、 概述 2
二、 心电图机介绍 2
三、 现场检查步骤 4
四、 数据导出和传输 5
五、 打印纸放置设置方式 5
六、 安全注意事项 6
七、 仪器的维护和清洁 6
八、 常见故障及处理 7
附录：心电图机中英文操作界面转换 8

一、 概述

心电图（electrocardiogram，ECG）是利用心电图机从体表记录心脏每一心动周期内产生电活动变化的曲线图形。临床上，心电图检查是诊断心律失常、冠心病等循环系统疾病的重要手段。

在中国慢性病前瞻性研究（CKB）的第二次重复调查中，将增加心电图的检查。在现场流程上，心电图检查将和血压、心率测量由同一个调查员先后完成，测量的具体顺序是先测心电图、然后测踝部血压，最后测上臂的血压和心率。本次调查中使用的心电图机是 Mortara ELI 250c。本文档将介绍仪器特点，使用和维护方法，现场检查步骤以及常见问题处理。

二、 心电图机介绍

（一）仪器简介

本次调查中使用的心电图机 Mortara ELI 250c 是一种 12 导联静态心电图机，可以用于采集、浏览、打印和储存心电图数据。仪器内置的心电图解译算法可以对心电图进行解析，并在心电图报告上打印分析结果。需提醒注意：仪器所提供的分析结果仅供参考，不能代替医学专业人士对心电图的分析和判断。取决于仪器自带软件的更新进度，此次调查中可能会要求使用英文界面以便于今后的数据分析。心电图机中英文操作界面转换操作步骤见附录。现场采集的心电图数据会自动储存在仪器中，每天现场调查的任务全部结束后，由数据传输负责人使用专门的其它设备 U 盘导出数据并通过项目办电脑按标准操作规程上传北京国家项目办和牛津国际项目办。

（二）仪器外观

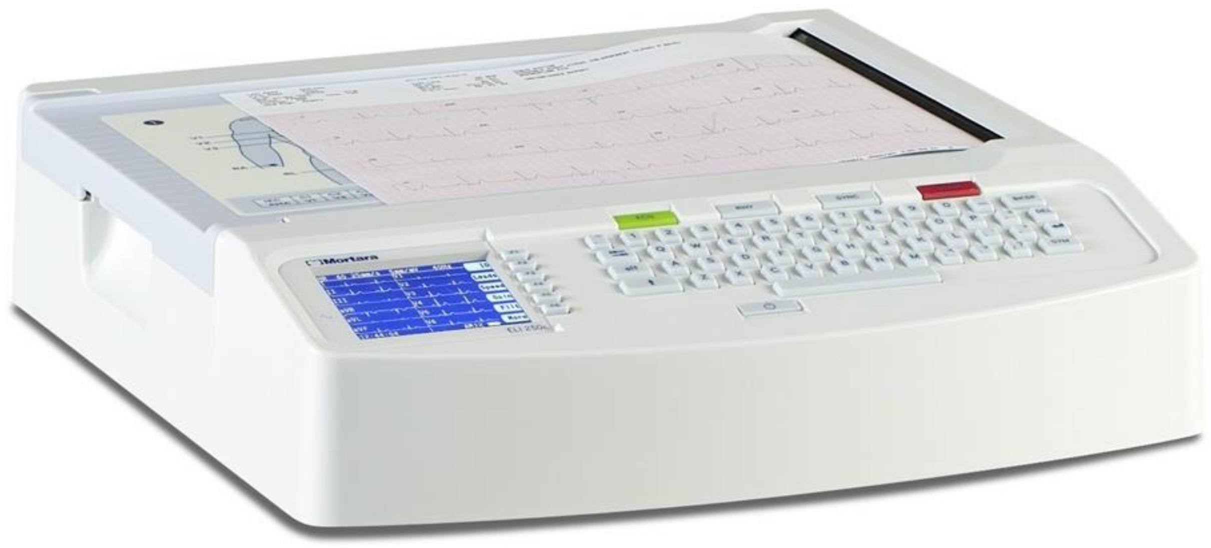

Mortara ELI 250c

（三）键盘和专用键

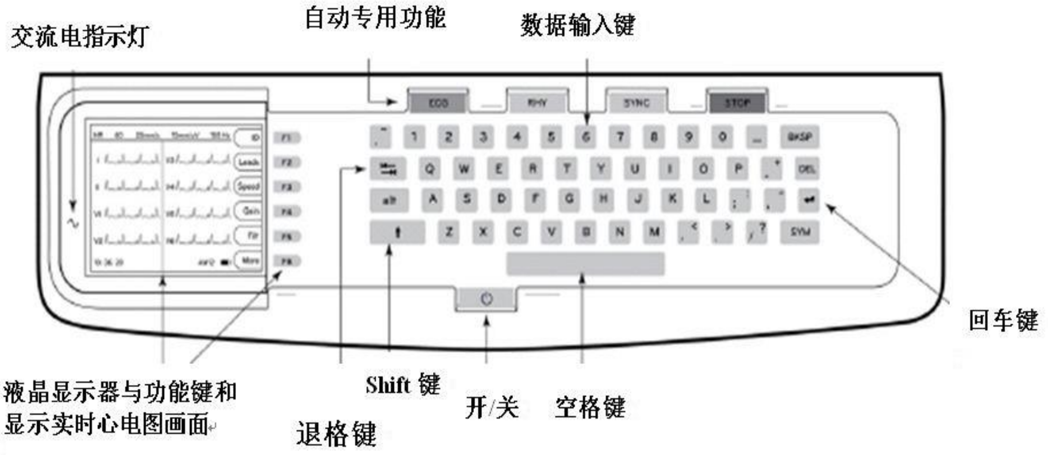

本仪器采用全键盘输入，键盘上的主要功能键如上图所示。键盘的最顶端有一排专用按钮，从左至右的功能分别是：

ECG：采集心电图

RHY：打印心率

SYNC：传输数据

STOP：停止

（四）显示器

本仪器配备了 320×240 像素的彩色液晶显示屏,可以显示心电图波形、功能键标签和其他参数。在心电图采集过程中，相应的参数会显示在屏幕上。在实时心电图界面下，屏幕显示如下图：

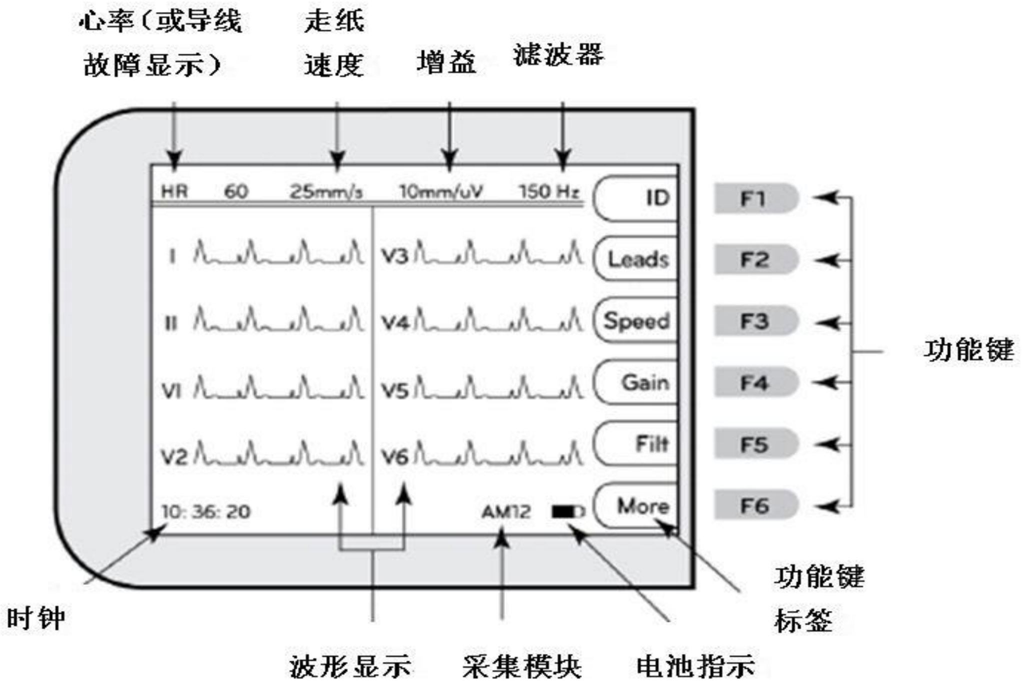

三、 现场检查步骤

本次调查中，心电图检查将在血压和心率测量之前由同一调查员执行。因为心电图检查需要被检查者袒露胸部，因此在检查时，应当在独立封闭的空间内进行，或拉上围帘、屏风等遮挡物，以保护被检查者隐私。现场检查步骤如下：

1. 检查心电图纸是否已放好（放置方法请参照本手册第 5 页），插上电源开机，此时屏幕上显示为实时心电图界面（如上图）。
2. 让调查对象平卧在检查床上，充分暴露其双侧手腕、脚踝和胸部，在导联连接部位涂抹一些温水。
3. 放置导联：

- 肢体导联：将肢体导联夹在调查对象的双侧手腕和脚踝处，使电极片位于身体内侧。红色导联夹在右手腕上，黄色导联夹在左手腕上，绿色导联夹在左脚踝上，黑色导联夹在右脚踝上。
- 胸导联：将胸导联吸附在涂抹过温水的皮肤上。6 个胸导联的放置位置如下表及图所示。放置胸导联时，可以先找到胸骨体与胸骨柄连接处的突起，此处平对第二肋间隙，向下可顺次找到第四和第五肋间隙。

导联	位置
**V1**	胸骨右缘第四肋间隙
**V2**	胸骨左缘第四肋间隙
**V3**	V2 和 V4 连线中点
**V4**	左锁骨中线上第五肋间隙
**V5**	左腋前线上 V4 水平
**V6**	左腋中线上 V4 水平

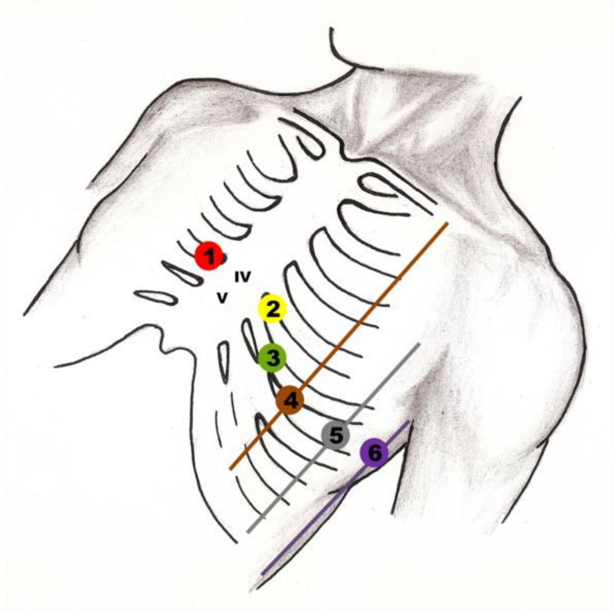

4. 按 F1 键，此时屏幕显示为调查对象的个人信息界面。使用条码扫描仪在此界面上输入调查员编码及调查对象的年龄、性别和第二次复查研究编码。输入位置如下：

   - First Name：调查员编码；
   - ID：调查对象的第二次复查研究编码（在《知情同意书》上可以找到）；

- Age：调查对象的年龄。可用键盘输入，也可以扫描邀请信上的年龄条码；
- Gender：调查对象的性别。男性输入 M，显示为 Male；女性输入 F，显示为 Female。

输入完成后，按 F6 确认，即可回到实时心电图界面。

5. 开始采集心电图：叮嘱调查对象不要说话和移动身体，放松肢体，平静呼吸。屏幕上可能出现提示语，提示导联连接脱落或连接位置错误。此时按照提示调整导联连接，确保导联正确连接。确认心电图图形平稳后，按下 ECG 键，屏幕上的实时心电图会被记录下的心电图画面取代，心电图被采集。
6. 测量结束后，所采集的数据将会自动保存。仪器将会自动打印出心电图报告。将导联从调查对象身上取下，并让调查对象继续保持平卧以测量踝部血压。心电图报告可以当场交给调查对象。

四、 数据导出和传输

每天现场调查结束后，由专人使用专用 U 盘（特殊设备 U 盘）将检查数据从仪器中导出，并进行数据同步。操作步骤如下：

1. 确认屏幕上显示的是实时心电图界面
2. 将 U 盘插入设备背部的 USB 插口中；
3. 按一下 SYNC 键；
4. 然后按一下 F1（Batch），选择批量传输，此时开始传输数据；
5. 传输成功后，屏幕上会显示“Transmission Completed. Press Stop to Continue”字样，意味着传输完成，此时应按 STOP 键，然后拔下 U 盘；
6. 回到地区项目办公室后将所保存的数据和其他数据一起按标准操作规程上传国家及牛津国际项目办。

五、 打印纸放置设置方式

仪器内置的热敏打印机可以即时打印出所采集的心电图。心电图机使用的打印用心电图纸由国家项目办统一提供。放置打印纸的步骤如下：

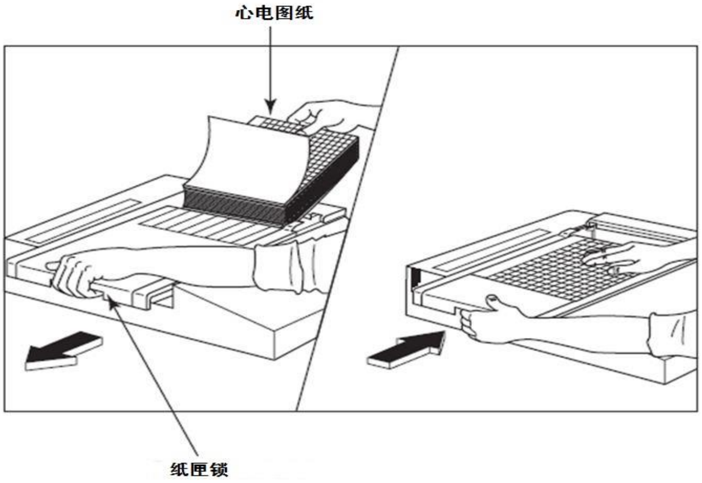

1. 将纸盒的外包装拆开。

2. 移动设备前方左侧的纸匣释放锁，向左滑动纸匣盖板。
3. 将一叠心电图纸放入纸匣，有网格的一面朝上，纸的提示标志（黑色小矩形）位于左下角。
4. 手动推送一页打印纸，确认纸已均匀地铺在位于纸匣通道内的黑色滚轴上。如果纸无法前进，可能发生卡纸故障。
5. 向右滑动盖板，直到听见一声清脆的滴答声，此时纸匣锁锁定。

六、 安全注意事项

使用心电图机时，请注意以下安全事项。更加详细的安全事项请参阅产品说明书和安全手册。

- 使用合适的电源线和保险丝，不要随意更换产品原装配件。
- 不要在存在可燃性气体的环境下使用仪器，这可能引起爆炸。
- 不要对仪器进行消毒，这可能损坏仪器。
- 不要在仪器上或旁边放置液体，防止液体洒到仪器上。
- 定期对仪器进行安全检查和维护。
- 停电时，请使用心电图机自带的锂电池供电，在充满状态下，锂电池可供 200 名调查对象使用，请勿使用柴油发电机供电，以免影响设备精准。
- 电池指示器在充电时无显示，当充电完全后显示白色。当电池电量低时，电池指示器呈绿色时表示 35%至 100%充电，呈黄色时表示 20%至 35%充电，当电池电量少于 20%时，电池指示器为红色。

七、 仪器的维护和清洁

（一）维护频率

仪器使用期间，应定期进行维护和清洁。国家项目办不要求对仪器内部进行任何处理，也不允许随意拆卸仪器。使用中只需对仪器的外部进行清洁和检查，并定期清洁打印喷头。

维护内容	维护频率	备注
外部检查和清洁	每周	外观有污物时随时清洁
清洁打印喷头	每天	如打印喷头堵塞需随时处理

（二）外部清洁和检查

1. 外部清洁：使用干布擦拭仪器的外壳和电源线。不要使用酒精、溶剂或其他清洁溶液。这些清洁剂可能损坏仪器的表面。
2. 外部检查：检查仪器外观是否有磨损、锈蚀、变质或坠落造成的损坏。

（三）清洁打印喷头步骤

1. 打开打印喷头盖；
2. 有沾有酒精的干净棉签轻轻擦拭打印喷头；

3. 用干净的干布擦拭喷头上的酒精；
4. 将喷头风干；
5. 用胶带清理滚筒。沾上胶带再撕下来。旋转滚筒，并重复这种清洁方式，直至滚筒干净；
6. 清洁完毕后，装回喷头盖。

八、 常见故障及处理

当仪器出现故障时，显示屏上会出现相应提示。按照下表中列举的方法处理这些故障，或联系国家项目办。

（一）仪器运行故障：

屏幕显示的信息	问题	解决方法
电池电量低，需充电	无法采集或打印心电图	用交流电给电池充电
导联故障，无法采集心电图	导联故障或心电图数据出现噪音	更换故障导联或采取降噪措施

（二）导联故障：

受影响的导联	问题	解决方法
任一导联断开	导联故障	检查导联的连接有否松动；更换故障导联。
导联 I	RA/LA 消失或噪音	检查患者准备工作是否进行得当；如需要可更换新的电极，重新进行准备工作。
导联 II	RA/LL 消失或噪音	检查患者准备工作是否进行得当；如需要可更换新的电极，重新进行准备工作。
导联 III	LA/LL 消失或噪音	检查患者准备工作是否进行得当；如需要可更换新的电极，重新进行准备工作。
全部	高频噪音	调整地滤波器设置；检查的电源线连接；检查交流滤波器（50Hz 或 150Hz）。

附录：心电图机中英文操作界面转换

<table>
<tr>
<td>1.　在实时心电图界面下按 F6；</td>
<td>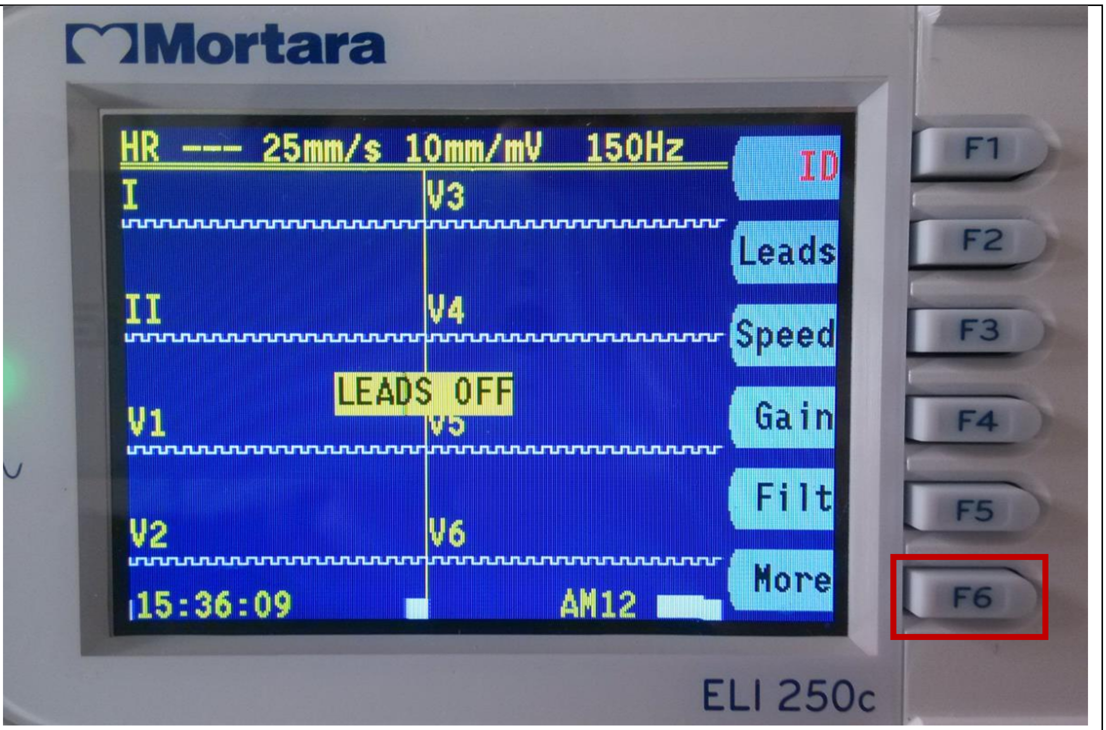
</td>
</tr>
<tr>
<td>2.　再按 F5；</td>
<td>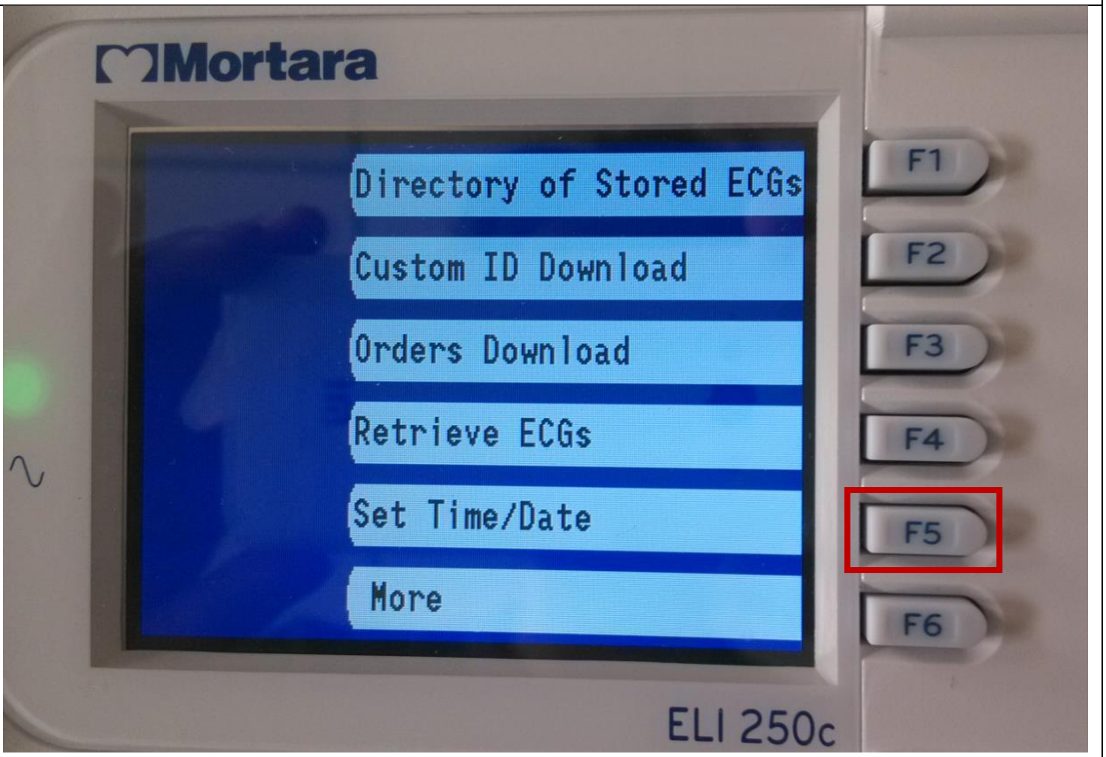
</td>
</tr>
</table>

3. 同时按下 F1 和 F2，输入“**admin**”；	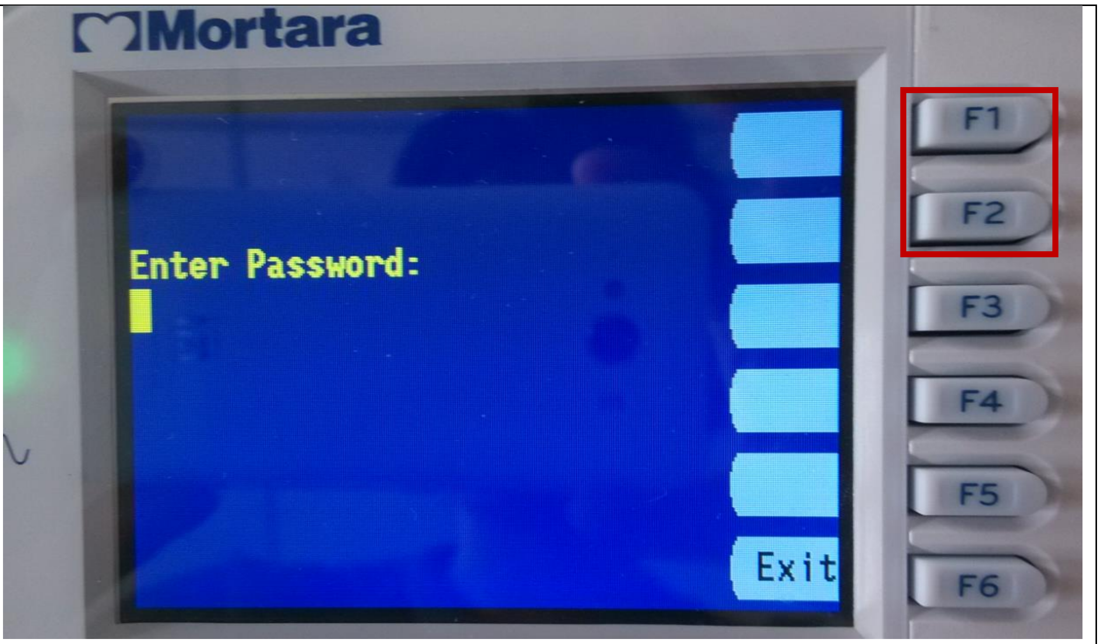
4. 将光标调至“语言”栏，按 F3 进行语言切换；“English”为英文制式，“Chinese”为中文制式。	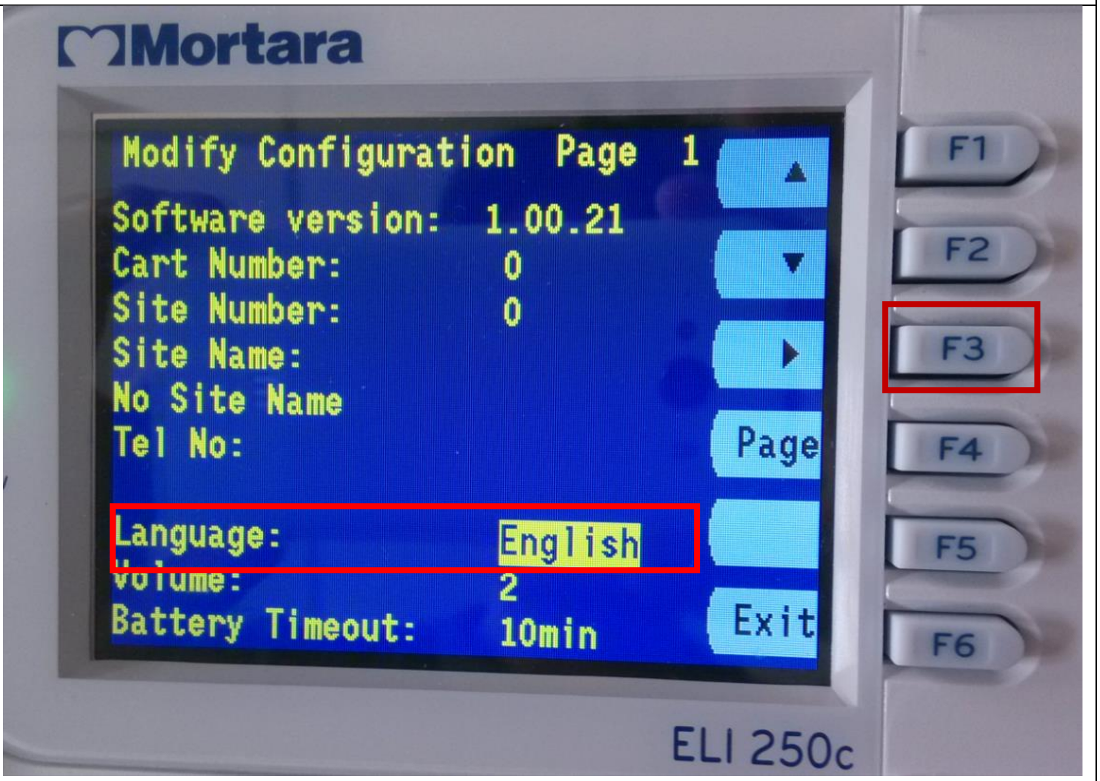
5. 按 F6 退出。	
