# Supplementary material for: Correlates and consequences of atrial fibrillation in a prospective study of 25 000 participants in the China Kadoorie Biobank
Source: Eur Heart J Open. 2024 Mar 19;4(2):oeae021. doi: 10.1093/ehjopen/oeae021 (PMC10989653; doi:10.1093/ehjopen/oeae021)
Supplement: oeae021_Supplementary_Data [file oeae021_supplementary_data.zip › Supplementary Material 22-3-2024.pdf]

Supplementary Material for "Correlates and consequences of atrial fibrillation in a prospective study of 25,000 participants in the China Kadoorie Biobank"

Table of contents

Members of the China Kadoorie Biobank Collaborative Group......................................2

Supplemental Methods S1A. Description of CHA_2DS_2-VASc score for Atrial Fibrillation Stroke Risk..3

Supplemental Methods S1B. Description of modified CHA_2DS_2-VASc score used in present report...4

Supplemental Methods S2: Descriptions of derived electrocardiographic (ECG) phenotypes...6

Table S1. Prevalence of clinical components of CHA_2DS_2-VASc score...................... 17

Table S2A. Prevalence of ECG phenotypes by age and CHA_2DS_2-VASc score in males only... 18

Table S2B. Prevalence of ECG phenotypes by age and CHA_2DS_2-VASc score in females...19

Table S3. Prevalence of ECG phenotypes by 5 urban and 5 rural areas.................... 20

Table S4. Prevalence of ECG phenotypes by age and CHA_2DS_2-VASc score without Suzhou area.. 21

Figure S1. Associations of ECG phenotypes with heart failure...................................22

Figure S2. Associations of three ECG phenotypes with stroke, CVD, heart failure and IHD, with and without Suzhou area... 23

Figure S3. Cumulative incidence of CVD for AF phenotype, only by rural vs urban ... 24

Figure S4. Cumulative incidence of CVD by sex...25

Figure S5. Cumulative incidence of CVD by status of self-reported prior CVD............26

Figure S6. Cumulative incidence of CVD with Suzhou area excluded.........................27

Members of the China Kadoorie Biobank Collaborative Group

International Steering Committee: Junshi Chen, Zhengming Chen (PI), Robert Clarke, Rory Collins, Liming Li (PI), Jun Lv, Richard Peto, Robin Walters.

International Co-ordinating Centre, Oxford: Daniel Avery, Derrick Bennett, Ruth Boxall, Ka Hung Chan, Yiping Chen, Zhengming Chen, Charlotte Clarke, Johnathan Clarke, Robert Clarke, Huaidong Du, Geoffrey Ma, Ahmed Edris Mohamed, Hannah Fry, Simon Gilbert, Pek Kei Im, Andri Iona, Maria Kakkoura, Christiana Kartsonaki, Hubert Lam, Kuang Lin, James Liu, Mohsen Mazidi, Iona Millwood, Sam Morris, Qu nhua Nie, Alfred Pozarickij, Maryam Rahmati, Paul Ryder, Dan Schmidt, Becky Stev ens, Iain Turnbull, Robin Walters, Baihan Wang, Lin Wang, Neil Wright, Ling Yang, Xiaoming Yang, Pang Yao.

National Co-ordinating Centre, Beijing: Xiao Han, Can Hou, Qingmei Xia, Chao Liu, Jun Lv, Pei Pei, Dianjianyi Sun, Canqing Yu, Lang Pan

10 Regional Co-ordinating Centres:
Guangxi Provincial CDC: Naying Chen, Duo Liu, Zhenzhu Tang. Liuzhou CDC: Ningyu Chen, Qilian Jiang, Jian Lan, Mingqiang Li, Yun Liu, Fanwen Meng, Jinhuai Meng, Rong Pan, Yulu Qin, Ping Wang, Sisi Wang, Liuping Wei, Liyuan Zhou. **Gansu** Provincial CDC: Caixia Dong, Pengfei Ge, Xiaolan Ren. Maiji CDC: Zhongxiao Li, Enke Mao, Tao Wang, Hui Zhang, Xi Zhang. **Hainan** Provincial CDC: Jinyan Chen, Ximin Hu, Xiaohuan Wang. Meilan CDC: Zhendong Guo, Huimei Li, Yilei Li, Min Weng, Shukuan Wu. **Heilongjiang** Provincial CDC: Shichun Yan, Mingyuan Zou, Xue Zhou. Nangang CDC: Ziyan Guo, Quan Kang, Yanjie Li, Bo Yu, Qinai Xu. **Henan** Provincial CDC: Liang Chang, Lei Fan, Shixian Feng, Ding Zhang, Gang Zhou. Huixian CDC: Yulian Gao, Tianyou He, Pan He, Chen Hu, Huarong Sun, Xukui Zhang. **Hunan** Provincial CDC: Biyun Chen, Zhongxi Fu, Yuelong Huang, Huilin Liu, Qiaohua Xu, Li Yin. Liuyang CDC: Huajun Long, Xin Xu, Hao Zhang, Libo Zhang. **Jiangsu** Provincial CDC: Jian Su, Ran Tao, Ming Wu, Jie Yang, Jinyi Zhou, Yonglin Zhou. Suzhou CDC: Yihe Hu, Yujie Hua, Jianrong Jin, Fang Liu, Jingchao Liu, Yan Lu, Liangcai Ma, Aiyu Tang, Jun Zhang. **Qingdao** CDC: Liang Cheng, Ranran Du, Ruqin Gao, Feifei Li, Shanpeng Li, Yongmei Liu, Feng Ning, Zengchang Pang, Xiaohui Sun, Xiaocao Tian, Shaojie Wang, Yaoming Zhai, Hua Zhang, Licang CDC: Wei Hou, Silu Lv, Junzheng Wang. **Sichuan** Provincial CDC: Xiaofang Chen, Xianping Wu, Ningmei Zhang, Weiwei Zhou. Pengzhou CDC: Xiaofang Chen, Jianguo Li, Jiaqiu Liu, Guojin Luo, Qiang Sun, Xunfu Zhong. **Zhejiang** Provincial CDC: Weiwei Gong, Ruying Hu, Hao Wang,Meng Wang, Min Yu. Tongxiang CDC: Lingli Chen, Qijun Gu, Dongxia Pan， Chunmei Wang, Kaixu Xie, Xiaoyi Zhang.

Supplementary Methods S1A: Description of CHA_2DS_2-VASc score for Atrial Fibrillation Stroke Risk

The CHA_2DS_2-VASc score comprised the following components:

- **Congestive heart failure / LV dysfunction**
  - no = 0
  - yes = 1
- **Hypertension**
  - no = 0
  - yes = 1
- **Age**
  - ≥75 years = 2
- **Diabetes mellitus**
  - no = 0
  - yes = 1
- **Stroke / TIA / TE**
  - no = 0
  - yes = 2
- **Vascular disease (prior myocardial infarction, peripheral artery disease, or aortic plaque)**
  - no = 0
  - yes = 1
- **Age**
  - <65 years = 0
  - 65 – 74 years = 1
- **Sex category**
  - female = 1
  - male = 0

LV = left ventricular; TIA = transient ischaemic attack; TE = thromboembolism

Table adapted from:

Refining clinical risk stratification for predicting stroke and thromboembolism in atrial fibrillation using a novel risk factor-based approach: the euro heart survey on atrial fibrillation.

Lip et al. *Chest*. 2010 Feb;137(2):263-72.

Supplementary methods S1B. Description of modified CHA_2DS_2-VASc score used in present report

The modified CHA_2DS_2-VASc score comprised the following components:

- **prior incident hospitalised episodes of heart failure**
  - no = 0
  - yes = 1

- **prior self-reported or physician-diagnosed hypertension or blood pressure measurement > 140/90 mmHg at resurvey**
  - no = 0
  - yes = 1

- **age at resurvey**
  - <65 years = 0
  - 65 – 74 years = 1
  - ≥75 years = 2

- **prior self-reported or physician-diagnosed diabetes mellitus or raised random plasma glucose***
  - no = 0
  - yes = 1

- **prior self-reported stroke or TIA**
  - no = 0
  - yes = 2

- **prior self-reported non-stroke CVD (acute MI, angina, or other IHD)**
  - no = 0
  - yes = 1

- **sex**
  - female = 1
  - male = 0

*diabetes detected by screening was defined as: (i) a random plasma glucose level ≥7.0 mmol/L, with time since last ate food of 8 hours or longer or ≥11.1 mmol/L with time since last ate of less than 8 hours or (ii) a fasting plasma glucose level of ≥7.0 mmol/) on subsequent testing.

TIA = transient ischaemic attack; CVD = cardiovascular disease; MI = myocardial infarction; IHD = ischaemic heart disease

Notes

The modified CHA_2DS_2-VASc score differed from the original scoring system in the following ways:

a) Save heart failure, for which all outcomes were hospitalised events, all relevant prior disease outcomes were self-reported by participants in questionnaires;
b) Participants with a blood pressure measurement > 140/90 mmHg at resurvey were regarded as having a diagnosis of hypertension;
c) Participants with raised random plasma glucose* at resurvey were regarded as having a diagnosis of diabetes mellitus;
d) Self-reported history of thromboembolism was not included in the modified scoring system; and,
e) Evidence of prior vascular disease was limited to self-reported ischaemic heart disease and did not include peripheral artery disease or aortic plaque.

Supplementary Methods S2: Descriptions of derived electrocardiographic (ECG) phenotypes used

ECG classification used in this report	ECG phenotype code	MORTARA diagnosis label
Unclassifiable	1	PEDIATRIC ECG INTERPRETATION
Unclassifiable	1	ABNORMAL ECG
Unclassifiable	1	ARM LEADS REVERSED
Unclassifiable	1	ATYPICAL ECG
Unclassifiable	1	BORDERLINE ECG
Unclassifiable	1	INTERPRETATION BASED ON A DEFAULT AGE OF 40 YEARS
Unclassifiable	1	NO FURTHER INTERPRETATION POSSIBLE
Unclassifiable	1	POSSIBLE ABNORMAL ECG
Unclassifiable	1	POSSIBLY ACUTE"
Unclassifiable	1	UNCERTAIN IRREGULAR RHYTHM
Unclassifiable	1	UNCERTAIN REGULAR RHYTHM
Unclassifiable	1	UNCONFIRMED REPORT
Unclassifiable	1	WARNING: DATA QUALITY MAY AFFECT INTERPRETATION
Unclassifiable	1	"TALL T-WAVES
Unclassifiable	1	ABNORMAL RISEN R WAVE
Unclassifiable	1	PROBABLY OLD WITH POSTERIOR EXTENSION [PROMINENT R WAVE IN V1/V2]"
Unclassifiable	1	PROBABLY OLD"
Unclassifiable	1	PROBABLY RECENT"
Normal/Normal Variant	2	CONSIDER NORMAL VARIANT"
Normal/Normal Variant	2	EARLY REPOLARIZATION
Normal/Normal Variant	2	NORMAL ECG
Normal/Normal Variant	2	NORMAL RANGE ECG
Normal/Normal Variant	2	OBVIOUS SINUS RHYTHM WITH SINUS ARRHYTHMIA
Normal/Normal Variant	2	OF INDETERMINATE AGE WITH POSTERIOR EXTENSION [PROMINENT R WAVE IN V1/V2]"
Normal/Normal Variant	2	OF INDETERMINATE AGE"

Normal/Normal Variant	2	OR EARLY REPOLARIZATION"
Normal/Normal Variant	2	OR NORMAL VARIANT"
Normal/Normal Variant	2	PROBABLY EARLY REPOLARIZATION"
Normal/Normal Variant	2	SINUS RHYTHM
Normal/Normal Variant	2	SINUS RHYTHM WITH MARKED SINUS ARRHYTHMIA
Normal/Normal Variant	2	SINUS RHYTHM WITH SINUS ARRHYTHMIA
Normal/Normal Variant	2	SINUS TACHYCARDIA
Ischaemic - Definite	3	"ANTERIOR MYOCARDIAL INFARCTION
Ischaemic - Definite	3	"ANTEROLATERAL MYOCARDIAL INFARCTION
Ischaemic - Definite	3	"ANTEROSEPTAL MYOCARDIAL INFARCTION
Ischaemic - Definite	3	"INFERIOR MYOCARDIAL INFARCTION
Ischaemic - Definite	3	"LATERAL MYOCARDIAL INFARCTION
Ischaemic - Definite	3	"MARKED ST DEPRESSION
Ischaemic - Definite	3	"MARKED ST ELEVATION
Ischaemic - Definite	3	"MARKED T-WAVE ABNORMALITY
Ischaemic - Definite	3	"SEPTAL MYOCARDIAL INFARCTION
Ischaemic - Definite	3	***ACUTE MI***
Ischaemic - Probable	4	"JUNCTIONAL ST DEPRESSION
Ischaemic - Probable	4	"MODERATE T-WAVE ABNORMALITY
Ischaemia - Probable	4	"ST DEVIATION AND MARKED T-WAVE ABNORMALITY
Ischaemia - Probable	4	"ST ELEVATION
Ischaemia - Probable	4	"ST ELEVATION CONSISTENT WITH INJURY
Ischaemia - Probable	4	MODERATE ST DEPRESSION
Ischaemic - Possible	5	"NEGATIVE PV1
Ischaemic - Possible	5	"POSSIBLE ANTERIOR MYOCARDIAL INFARCTION
Ischaemic - Possible	5	"POSSIBLE ANTEROLATERAL MYOCARDIAL INFARCTION
Ischaemic - Possible	5	"POSSIBLE ANTEROSEPTAL MYOCARDIAL INFARCTION
Ischaemic - Possible	5	"POSSIBLE INFERIOR MYOCARDIAL INFARCTION
Ischaemic - Possible	5	"POSSIBLE LATERAL MYOCARDIAL INFARCTION
Ischaemic - Possible	5	"POSSIBLE SEPTAL MYOCARDIAL INFARCTION

Ischaemic - Probable	5	"PROBABLE ANTERIOR MYOCARDIAL INFARCTION
Ischaemic - Probable	5	"PROBABLE ANTEROLATERAL MYOCARDIAL INFARCTION
Ischaemic - Probable	5	"PROBABLE ANTEROSEPTAL MYOCARDIAL INFARCTION
Ischaemic - Probable	5	"PROBABLE INFERIOR MYOCARDIAL INFARCTION
Ischaemic - Probable	5	"PROBABLE LATERAL MYOCARDIAL INFARCTION
Ischaemic - Probable	5	"PROBABLE SEPTAL MYOCARDIAL INFARCTION
Ischaemia - Possible	5	"ST DEPRESSION
Ischaemia - Possible	5	"ST DEVIATION AND MODERATE T-WAVE ABNORMALITY
Ischaemia - Possible	5	BORDERLINE ST DEPRESSION
Ischaemia - Possible	5	CONSIDER SUBENDOCARDIAL INJURY"
Ischaemia - Possible	5	CONSIDER ANTERIOR INJURY"
Ischaemia - Possible	5	CONSIDER ANTERIOR ISCHEMIA"
Ischaemia - Possible	5	CONSIDER ANTEROLATERAL INJURY"
Ischaemia - Possible	5	CONSIDER ANTEROLATERAL ISCHEMIA"
Ischaemia - Possible	5	CONSIDER ANTEROSEPTAL INJURY"
Ischaemia - Possible	5	CONSIDER INFERIOR INJURY"
Ischaemia - Possible	5	CONSIDER INFERIOR ISCHEMIA"
Ischaemia - Possible	5	CONSIDER LATERAL INJURY"
Ischaemia - Possible	5	CONSIDER LATERAL ISCHEMIA"
Ischaemia - Possible	5	CONSIDER SEPTAL INJURY"
Ischaemia - Possible	5	CONSIDER SUBENDOCARDIAL INJURY"
Ischaemia - Possible	5	MINIMAL ST DEPRESSION
Ischaemia - Possible	5	NONSPECIFIC ST & T-WAVE ABNORMALITY
Ischaemia - Possible	5	NONSPECIFIC ST ELEVATION
Ischaemia - Possible	5	NONSPECIFIC T-WAVE ABNORMALITY
Ischaemia - Possible	5	ST-T CHANGE (BORDERLINE)
Ischaemia - Possible	5	ST-T CHANGE (POSSIBLE CARDIAC ISCHEMIA)
Ischaemia - Possible	5	T-WAVE CHANGE
Ischaemia - Possible	5	T-WAVE CHANGE 9LOW AND FLAT)
Arrhythmia - Atrial Fibrillation/Flutter	6	ATRIAL FIBRILLATION

Arrhythmia - Atrial Fibrillation/Flutter	6	ATRIAL FIBRILLATION (TACHYCARDIA)
Arrhythmia - Atrial Fibrillation/Flutter	6	ATRIAL FIBRILLATION WITH ABERRANT CONDUCTION OR VENTRICULAR PREMATURE COMPLEXES
Arrhythmia - Atrial Fibrillation/Flutter	6	ATRIAL FIBRILLATION WITH RAPID VENTRICULAR RESPONSE
Arrhythmia - Atrial Fibrillation/Flutter	6	ATRIAL FIBRILLATION WITH RAPID VENTRICULAR RESPONSE WITH ABERRANT CONDUCTION OR VENTRICULAR PREMATUR
Arrhythmia - Atrial Fibrillation/Flutter	6	ATRIAL FIBRILLATION WITH SLOW VENTRICULAR RESPONSE
Arrhythmia - Atrial Fibrillation/Flutter	6	ATRIAL FLUTTER/TACHYCARDIA
Arrhythmia - Atrial Fibrillation/Flutter	6	ATRIAL FLUTTER/TACHYCARDIA WITH RAPID VENTRICULAR RESPONSE
Arrhythmia - Atrial Fibrillation/Flutter	6	ATRIAL FLUTTER/TACHYCARDIA WITH SLOW VENTRICULAR RESPONSE WITH ABERRANT CONDUCTION OR VENTRICULAR PR
Arrhythmia - Atrial Fibrillation/Flutter	6	POSSIBLE ATRIAL FLUTTER"
Arrhythmia - Bradycardia/Heart block	7	JUNCTIONAL BRADYCARDIA
Arrhythmia - Bradycardia/Heart block	7	JUNCTIONAL RHYTHM
Arrhythmia - Bradycardia/Heart block	7	JUNCTIONAL RHYTHM WITH OCCASIONAL SUPRAVENTRICULAR PREMATURE COMPLEXES
Arrhythmia - Bradycardia/Heart block	7	JUNCTIONAL RHYTHM WITH OCCASIONAL VENTRICULAR PREMATURE COMPLEXES
Arrhythmia - Bradycardia/Heart block	7	ELECTRONIC ATRIAL PACEMAKER
Arrhythmia - Bradycardia/Heart block 7		ELECTRONIC VENTRICULAR PACEMAKER
Arrhythmia - Bradycardia/Heart block	7	ELECTRONIC VENTRICULAR PACEMAKER -- CONTOUR ANALYSIS BASED ON INTRINSIC RHYTHM
Arrhythmia - Bradycardia/Heart block	8	ECTOPIC ATRIAL BRADYCARDIA
Arrhythmia - Other arrhythmia/pro-arrhythmic condition	8	ECTOPIC ATRIAL RHYTHM
Arrhythmia - Other arrhythmia/pro-arrhythmic condition	8	ECTOPIC ATRIAL RHYTHM WITH FREQUENT VENTRICULAR PREMATURE COMPLEXES IN A BIGEMINAL PATTERN
Arrhythmia - Other arrhythmia/pro-arrhythmic condition	8	ECTOPIC ATRIAL RHYTHM WITH OCCASIONAL SUPRAVENTRICULAR PREMATURE COMPLEXES
Arrhythmia - Other arrhythmia/pro-arrhythmic condition	8	ECTOPIC ATRIAL RHYTHM WITH OCCASIONAL VENTRICULAR PREMATURE COMPLEXES

Arrhythmia - Other arrhythmia/pro-arrhythmic condition	8	ECTOPIC ATRIAL RHYTHM WITH PROLONGED PR INTERVAL
Arrhythmia - Other arrhythmia/pro-arrhythmic condition	8	ECTOPIC ATRIAL RHYTHM WITH SHORT PR INTERVAL
Arrhythmia - Other arrhythmia/pro-arrhythmic condition	8	ECTOPIC ATRIAL RHYTHM WITH SHORT PR INTERVAL WITH FREQUENT SUPRAVENTRICULAR PREMATURE COMPLEXES
Arrhythmia - Tachycardia	8	ECTOPIC ATRIAL TACHYCARDIA
Arrhythmia - Other arrhythmia/pro-arrhythmic condition	8	FREQUENT VENTRICULAR PREMATURE COMPLEXES
Arrhythmia - Other arrhythmia/pro-arrhythmic condition	8	INTERMITTENT VENTRICULAR PREEXCITATION/WPW
Arrhythmia - Tachycardia	8	JUNCTIONAL TACHYCARDIA
Arrhythmia - Other arrhythmia/pro-arrhythmic condition	8	PROLONGED QT INTERVAL
Arrhythmia - Other arrhythmia/pro-arrhythmic condition	8	QTc SHORTEN
Arrhythmia - Other arrhythmia/pro-arrhythmic condition	8	QTs DELAYED
Arrhythmia - Tachycardia	8	"ECTOPIC ATRIAL TACHYCARDIA
Arrhythmia - Bradycardia/Heart block 8		"SINUS BRADYCARDIA WITH 2ND DEGREE AV BLOCK
Arrhythmia - Bradycardia/Heart block 8		"SINUS RHYTHM WITH 2ND DEGREE AV
BLOCK Arrhythmia - Other arrhythmia/pro-arrhythmic condition	8	"SINUS RHYTHM WITH MARKED RHYTHM IRREGULARITY
Arrhythmia - Bradycardia/Heart block 8		"SINUS TACHYCARDIA WITH 2ND DEGREE AV BLOCK
Arrhythmia - Other arrhythmia/pro-arrhythmic condition	8	ABNORMAL RHYTHM ECG
Arrhythmia - Bradycardia/Heart block	8	AV BLOCK
Arrhythmia - Other arrhythmia/pro-arrhythmic condition	8	OCCASIONAL ATRIAL PREMATURE COMPLEXES
Arrhythmia - Other arrhythmia/pro-arrhythmic condition	8	OCCASIONAL VENTRICULAR PREMATURE COMPLEXES
Arrhythmia - Bradycardia/Heart block	8	OR SINUS PAUSE"

Arrhythmia - Other arrhythmia/pro-arrhythmic condition	8	POSSIBLE NON-CONDUCTED PAC
Arrhythmia - Bradycardia/Heart block	8	SA BLOCK
Arrhythmia - Other arrhythmia/pro-arrhythmic condition	8	SHORTEN PR INTERVAL
Arrhythmia - Bradycardia/Heart block	8	SINUS BRADYCARDIA
Arrhythmia - Bradycardia/Heart block	8	SINUS BRADYCARDIA WITH FREQUENT SUPRAVENTRICULAR PREMATURE COMPLEXES
Arrhythmia - Bradycardia/Heart block 8		SINUS BRADYCARDIA WITH MARKED SINUS ARRHYTHMIA
Arrhythmia - Bradycardia/Heart block 8		SINUS BRADYCARDIA WITH OCCASIONAL ECTOPIC PREMATURE COMPLEXES
Arrhythmia - Bradycardia/Heart block		SINUS BRADYCARDIA WITH OCCASIONAL SUPRAVENTRICULAR PREMATURE COMPLEXES
Arrhythmia	8	SINUS BRADYCARDIA WITH OCCASIONAL VENTRICULAR PREMATURE COMPLEXES
Arrhythmia - Bradycardia/Heart block		SINUS BRADYCARDIA WITH OCCASIONAL VENTRICULAR PREMATURE COMPLEXES WITH FREQUENT SUPRAVENTRICULAR PRE
Arrhythmia - Bradycardia/Heart block 8		SINUS BRADYCARDIA WITH PROLONGED PR INTERVAL
Arrhythmia - Bradycardia/Heart block 8		SINUS BRADYCARDIA WITH PROLONGED PR INTERVAL WITH OCCASIONAL SUPRAVENTRICULAR PREMATURE COMPLEXES
Arrhythmia - Bradycardia/Heart block 8		SINUS BRADYCARDIA WITH SHORT PR INTERVAL
Arrhythmia - Bradycardia/Heart block 8		SINUS BRADYCARDIA WITH SINUS ARRHYTHMIA
Arrhythmia - Bradycardia/Heart block	8	SINUS BRADYCARDIA WITH SINUS ARRHYTHMIA WITH PROLONGED PR INTERVAL
Arrhythmia - Bradycardia/Heart block 8		SINUS BRADYCARDIA WITH SINUS ARRHYTHMIA WITH SHORT PR
INTERVAL Arrhythmia - Other arrhythmia/pro-arrhythmic condition	8	SINUS RHYTHM WITH FREQUENT ECTOPIC PREMATURE COMPLEXES
Arrhythmia - Other arrhythmia/pro-arrhythmic condition	8	SINUS RHYTHM WITH FREQUENT ECTOPIC PREMATURE COMPLEXES IN A BIGEMINAL PATTERN
Arrhythmia - Other arrhythmia/pro-arrhythmic condition	8	SINUS RHYTHM WITH FREQUENT SUPRAVENTRICULAR PREMATURE COMPLEXES
Arrhythmia - Other arrhythmia/pro-arrhythmic condition	8	SINUS RHYTHM WITH FREQUENT SUPRAVENTRICULAR PREMATURE COMPLEXES IN A BIGEMINAL PATTERN

Arrhythmia - Other arrhythmia/pro-arrhythmic condition	8	SINUS RHYTHM WITH FREQUENT VENTRICULAR PREMATURE COMPLEXES
Arrhythmia - Other arrhythmia/pro-arrhythmic condition	8	SINUS RHYTHM WITH FREQUENT VENTRICULAR PREMATURE COMPLEXES IN A BIGEMINAL PATTERN
Arrhythmia - Bradycardia/Heart block	8	SINUS RHYTHM WITH HIGH GRADE AV BLOCK
Arrhythmia - Bradycardia/Heart block	8	SINUS RHYTHM WITH MARKED SINUS ARRHYTHMIA WITH PROLONGED PR INTERVAL
Arrhythmia - Other arrhythmia/pro-arrhythmic condition	8	SINUS RHYTHM WITH MARKED SINUS ARRHYTHMIA WITH SHORT PR INTERVAL
Arrhythmia - Other arrhythmia/pro-arrhythmic condition	8	SINUS RHYTHM WITH OCCASIONAL ECTOPIC PREMATURE COMPLEXES
Arrhythmia - Other arrhythmia/pro-arrhythmic condition	8	SINUS RHYTHM WITH OCCASIONAL SUPRAVENTRICULAR PREMATURE COMPLEXES
Arrhythmia - Other arrhythmia/pro-arrhythmic condition	8	SINUS RHYTHM WITH OCCASIONAL VENTRICULAR PREMATURE COMPLEXES
Arrhythmia - Other arrhythmia/pro-arrhythmic condition	8	SINUS RHYTHM WITH OCCASIONAL VENTRICULAR PREMATURE COMPLEXES WITH FREQUENT SUPRAVENTRICULAR PREMATUR
Arrhythmia - Other arrhythmia/pro-arrhythmic condition	8	SINUS RHYTHM WITH OCCASIONAL VENTRICULAR PREMATURE COMPLEXES WITH OCCASIONAL SUPRAVENTRICULAR PREMAT
Arrhythmia - Bradycardia/Heart block	8	SINUS RHYTHM WITH PROLONGED PR INTERVAL
Arrhythmia - Bradycardia/Heart block	8	SINUS RHYTHM WITH PROLONGED PR INTERVAL WITH FREQUENT SUPRAVENTRICULAR PREMATURE COMPLEXES
Arrhythmia - Bradycardia/Heart block	8	SINUS RHYTHM WITH PROLONGED PR INTERVAL WITH FREQUENT VENTRICULAR PREMATURE COMPLEXES
Arrhythmia - Bradycardia/Heart block	8	SINUS RHYTHM WITH PROLONGED PR INTERVAL WITH FREQUENT VENTRICULAR PREMATURE COMPLEXES IN A BIGEMINAL
Arrhythmia - Bradycardia/Heart block	8	SINUS RHYTHM WITH PROLONGED PR INTERVAL WITH OCCASIONAL SUPRAVENTRICULAR PREMATURE COMPLEXES
Arrhythmia - Bradycardia/Heart block	8	SINUS RHYTHM WITH PROLONGED PR INTERVAL WITH OCCASIONAL VENTRICULAR PREMATURE COMPLEXES
Arrhythmia - Bradycardia/Heart block	8	SINUS RHYTHM WITH PROLONGED PR INTERVAL WITH OCCASIONAL VENTRICULAR PREMATURE COMPLEXES WITH OCCASIO

Arrhythmia - Other arrhythmia/pro-arrhythmic condition	8	SINUS RHYTHM WITH SHORT PR INTERVAL
Arrhythmia - Other arrhythmia/pro-arrhythmic condition	8	SINUS RHYTHM WITH SHORT PR INTERVAL WITH FREQUENT SUPRAVENTRICULAR PREMATURE COMPLEXES
Arrhythmia - Other arrhythmia/pro-arrhythmic condition	8	SINUS RHYTHM WITH SHORT PR INTERVAL WITH FREQUENT VENTRICULAR PREMATURE COMPLEXES
Arrhythmia - Other arrhythmia/pro-arrhythmic condition	8	SINUS RHYTHM WITH SHORT PR INTERVAL WITH OCCASIONAL ECTOPIC PREMATURE COMPLEXES
Arrhythmia - Other arrhythmia/pro-arrhythmic condition	8	SINUS RHYTHM WITH SHORT PR INTERVAL WITH OCCASIONAL SUPRAVENTRICULAR PREMATURE COMPLEXES
Arrhythmia - Other arrhythmia/pro-arrhythmic condition	8	SINUS RHYTHM WITH SHORT PR INTERVAL WITH OCCASIONAL VENTRICULAR PREMATURE COMPLEXES
Arrhythmia - Other arrhythmia/pro-arrhythmic condition	8	SINUS RHYTHM WITH SHORT PR INTERVAL WITH OCCASIONAL VENTRICULAR PREMATURE COMPLEXES WITH OCCASIONAL
Arrhythmia - Bradycardia/Heart block	8	SINUS RHYTHM WITH SINUS ARRHYTHMIA WITH PROLONGED PR INTERVAL
Arrhythmia - Other arrhythmia/pro-arrhythmic condition	8	SINUS RHYTHM WITH SINUS ARRHYTHMIA WITH SHORT PR INTERVAL
Arrhythmia - Other arrhythmia/pro-arrhythmic condition	8	SINUS TACHYCARDIA WITH FREQUENT ECTOPIC PREMATURE COMPLEXES
Arrhythmia - Other arrhythmia/pro-arrhythmic condition	8	SINUS TACHYCARDIA WITH FREQUENT SUPRAVENTRICULAR PREMATURE COMPLEXES
Arrhythmia - Other arrhythmia/pro-arrhythmic condition	8	SINUS TACHYCARDIA WITH FREQUENT VENTRICULAR PREMATURE COMPLEXES
Arrhythmia - Other arrhythmia/pro-arrhythmic condition	8	SINUS TACHYCARDIA WITH OCCASIONAL ECTOPIC PREMATURE COMPLEXES
Arrhythmia - Other arrhythmia/pro-arrhythmic condition	8	SINUS TACHYCARDIA WITH OCCASIONAL SUPRAVENTRICULAR PREMATURE COMPLEXES
Arrhythmia - Other arrhythmia/pro-arrhythmic condition	8	SINUS TACHYCARDIA WITH OCCASIONAL VENTRICULAR PREMATURE COMPLEXES
Arrhythmia - Other arrhythmia/pro-arrhythmic condition	8	SINUS TACHYCARDIA WITH OCCASIONAL VENTRICULAR PREMATURE COMPLEXES WITH OCCASIONAL SUPRAVENTRICULAR P
Arrhythmia - Bradycardia/Heart block	8	SINUS TACHYCARDIA WITH PROLONGED PR INTERVAL

Arrhythmia - Bradycardia/Heart block 8		SINUS TACHYCARDIA WITH PROLONGED PR INTERVAL WITH OCCASIONAL SUPRAVENTRICULAR PREMATURE
COMPLEXES Arrhythmia - Other arrhythmia/pro-arrhythmic condition	8	SINUS TACHYCARDIA WITH SHORT PR INTERVAL
Arrhythmia - Other arrhythmia/pro-arrhythmic condition	8	SINUS TACHYCARDIA WITH SHORT PR INTERVAL WITH FREQUENT SUPRAVENTRICULAR PREMATURE COMPLEXES
Arrhythmia - Other arrhythmia/pro-arrhythmic condition	8	SINUS TACHYCARDIA WITH SHORT PR INTERVAL WITH OCCASIONAL SUPRAVENTRICULAR PREMATURE COMPLEXES
Arrhythmia - Bradycardia/Heart block 8		SUPRAVENTRICULAR BRADYCARDIA
Arrhythmia - Other arrhythmia/pro-arrhythmic condition	8	SUPRAVENTRICULAR RHYTHM
Arrhythmia - Tachycardia	8	SUPRAVENTRICULAR TACHYCARDIA
Arrhythmia - Other arrhythmia/pro-arrhythmic condition DIAGNOSTIC)	8	TYPE 2 BRUGADA PATTERN (NON-
Arrhythmia - Other arrhythmia/pro-arrhythmic condition DIAGNOSTIC)	8	TYPE 3 BRUGADA PATTERN (NON-
Arrhythmia - Other arrhythmia/pro-arrhythmic condition	8	VENTRICULAR PREEXCITATION/WPW
Arrhythmia - Bradycardia/Heart block	8	MOBITZ TYPE I (WENCKEBACH)"
Arrhythmia - Bradycardia/Heart block	8	MOBITZ TYPE II"
LV Hypertrophy	9	HIGH VOLTAGE IN LEFT VENTRICAL
LV Hypertrophy	9	LEFT VENTRICULAR HYPERTROPHY AND ST-T CHANGE
LV Hypertrophy	9	VOLTAGE CRITERIA FOR LVH
LV Hypertrophy	10	"MODERATE VOLTAGE CRITERIA FOR LVH
LV Hypertrophy	11	"MINIMAL VOLTAGE CRITERIA FOR LVH
Other pathology	11	POSSIBLE LEFT VENTRICULAR HYPERTROPHY
Other pathology	12	"S1-S2-S3 PATTERN
Other pathology	12	"WIDEN QRS
Other pathology	12	ABNORMAL QRS-T ANGLE
Other pathology	12	BORDERLINE LEFT AXIS DEVIATION
Other pathology	12	BORDERLINE RIGHT AXIS DEVIATION

Other pathology	12	CONSISTENT WITH PULMONARY DISEASE
Other pathology	12	COUNTERCLOCKWISE ROTATION
Other pathology	12	DEXTROCARDIA
Other pathology	12	INCOMPLETE RIGHT BUNDLE BRANCH BLOCK
Other pathology	12	INDETERMINATE AXIS
Other pathology	12	INDICATING ENLARGED LEFT ATRIUM"
Other pathology	12	INTRAVENTRICULAR CONDUCTION DELAY
Other pathology	12	LEFT ANTERIOR FASCICULAR BLOCK
Other pathology	12	LEFT ATRIAL ENLARGEMENT
Other pathology	12	LEFT AXIS DEVIATION
Other pathology	12	LEFT BUNDLE BRANCH BLOCK
Other pathology	12	LEFT POSTERIOR FASCICULAR BLOCK
Other pathology	12	LOW QRS VOLTAGE
Other pathology	12	LOW QRS VOLTAGE IN EXTREMITY LEADS
Other pathology	12	LOW QRS VOLTAGE IN PRECORDIAL LEADS
Other pathology	12	LOW VOLTAGE (LIMB LEAD)
Other pathology	12	MARKED LEFT AXIS DEVIATION
Other pathology	12	MARKED RIGHT AXIS DEVIATION
Other pathology	12	MODERATE INTRAVENTRICULAR CONDUCTION DELAY
Other pathology	12	OBVIOUS COUNTERCLOCKWISE ROTATION
Other pathology	12	OBVIOUS LEFT AXIS DEVIATION
Other pathology	12	PATTERN CONSISTENT WITH PULMONARY DISEASE
Other pathology	12	PERICARDITIS
Other pathology	12	POSSIBLE INTRA-VENTRICULAR BLOCK"
Other pathology	12	POSSIBLE LEFT ATRIAL ENLARGEMENT
Other pathology	12	POSSIBLE RIGHT ATRIAL ENLARGEMENT
Other pathology	12	POSSIBLE RIGHT VENTRICULAR CONDUCTION DELAY
Other pathology	12	POSSIBLE RIGHT VENTRICULAR HYPERTROPHY
Other pathology	12	PROBABLE RIGHT VENTRICULAR HYPERTROPHY
Other pathology	12	RIGHT ATRIAL ENLARGEMENT

Other pathology	12	RIGHT AXIS DEVIATION
Other pathology	12	RIGHT BUNDLE BRANCH BLOCK
Other pathology	12	RIGHT BUNDLE BRANCH BLOCK AND POSSIBLE RIGHT VENTRICULAR HYPERTROPHY
Other pathology	12	RIGHT VENTRICULAR HYPERTROPHY
Other pathology	12	RIGHT VENTRICULAR HYPERTROPHY AND ST-T CHANGE
Other pathology	12	RVH
Other Pathology	12	SUGGESTS HYPERKALEMIA"

For arrhythmias, text strings were assigned to specific subcategories based on their descriptions. Text strings suggestive of arrhythmia were allocated up to two arrhythmic phenotypes. An independent phenotypic category of pro-atrial fibrillation (AF) condition was also established comprising clinically relevant ECG features, including the presence in text strings of PR interval prolongation or left and/or right atrial enlargement. Text strings for definite ischaemia included those with marked ST segment depression or elevation and/or marked T wave abnormalities and those with ECG features consistent with diagnosis of myocardial infarction. Probable ischaemia was characterised by ST segment elevation not meeting the criteria for definite ischaemia, moderate ST segment depression, or ST segment deviation accompanied by marked T wave abnormalities. The possible ischaemia phenotype consisted of text strings with minimal, borderline, or unspecified ST segment depression, nonspecific or borderline ST segment elevation and/or T wave abnormalities, ST segment deviation accompanied by moderate T wave abnormalities, or ECG features suggestive of transmural or sub-endocardial myocardial infarction or ischaemia. The left ventricular hypertrophy (LVH) phenotype contained text strings for minimal-, moderate-, or high-voltage Sokolov-Lyon criteria for LVH, with or without an LV strain pattern.

Table S1. Prevalence of clinical components of CHA_2DS_2-VASc score

	Score								
	**0**	**1**	**2**	**3**	**4**	**5**	**6**	**7**	**All**
**Number of participants**	3356	9374	6671	3538	1668	493	118	21	25239
**Medical history† , N(%)**									
Heart failure	0 (0.0)	2 (0.0)	2 (0.0)	12 (0.3)	12 (0.7)	4 (0.8)	7 (5.9)	2 (9.5)	41 (0.2)
Hypertension	0 (0.0)	2298 (24.5)	5095 (76.4)	3087 (87.3)	1587 (95.1)	479 (97.2)	117 (99.2)	21 (100.0)	12684 (50.3)
Diabetes	0 (0.0)	199 (2.1)	608 (9.1)	807 (22.8)	512 (30.7)	239 (48.5)	71 (60.2)	20 (95.2)	2456 (9.7)
Stroke/TIA	0 (0.0)	0 (0.0)	33 (0.5)	190 (5.4)	298 (17.9)	226 (45.8)	88 (74.6)	20 (95.2)	855 (3.4)
Non-stroke CVD	0 (0.0)	69 (0.7)	288 (4.3)	445 (12.6)	334 (20.0)	162 (32.9)	29 (24.6)	1 (4.8)	1328 (5.3)

† Prior diseases all self-reported except heart failure, which is reported from hospitalised episodes, hypertension, which includes those self-reported disease and those with SBP ≥ 140mmHg and DBP ≥ 90mmHg at resurvey, and diabetes, which includes self-reported disease and those with raised RPG at resurvey.
Percentages are calculated separately within columns.
ECG = electrocardiogram; CVD = cardiovascular disease; TIA = transient ischaemic attack; SBP = systolic blood pressure; DBP = diastolic blood pressure; RPG = random plasma glucose.

Table S2A. Prevalence of ECG phenotypes by age and CHA_2DS_2-VASc score in males only

	Age (years) at ECG measurement			CHA_2DS_2-VASc		
**ECG phenotypes**	**<55**	**55-69**	**70+**	**<2**	**2+**	**All**
**Number of participants**	3308	4439	1922	6564	3105	9669
**Arrhythmia / Pro-arrhythmia, N(%)**						
Atrial fibrillation / flutter	15 (0.5)	40 (0.9)	97 (5.0)	43 (0.7)	109 (3.5)	152 (1.6)
Pro-AF condition*	53 (1.6)	133 (3.0)	126 (6.6)	123 (1.9)	189 (6.1)	312 (3.2)
Other arrhythmia / tachycardia	196 (5.9)	354 (8.0)	304 (15.8)	453 (6.9)	401 (12.9)	854 (8.8)
Bradycardia / heart block	226 (6.8)	414 (9.3)	232 (12.1)	545 (8.3)	327 (10.5)	872 (9.0)
**Ischaemia, N(%)**						
Possible / probable	548 (16.6)	928 (20.9)	510 (26.5)	1108 (16.9)	878 (28.3)	1986 (20.5)
Definite	84 (2.5)	149 (3.4)	84 (4.4)	178 (2.7)	139 (4.5)	317 (3.3)
**Left ventricular hypertrophy, N(%)**	542 (16.4)	896 (20.2)	401 (20.9)	1196 (18.2)	643 (20.7)	1839 (19.0)
**Non-specific ECG changes, N(%)**	581 (17.6)	973 (21.9)	621 (32.3)	1287 (19.6)	888 (28.6)	2175 (22.5)
**Normal variant, N(%)**	1528 (46.2)	1716 (38.7)	507 (26.4)	2882 (43.9)	869 (28.0)	3751 (38.8)

Percentages are calculated separately within columns.
ECG = electrocardiogram; AF = atrial fibrillation.
*Pro-AF condition = phenotype comprising ECG features that may increase the risk of development of atrial fibrillation.

Table S2B. Prevalence of ECG phenotypes by age and CHA_2DS_2-VASc score in females

	Age (years) at ECG measurement			CHA_2DS_2-VASc		
**ECG phenotypes**	**<55**	**55-69**	**70+**	**<3**	**3+**	**All**
**Number of participants**	5841	7290	2439	11073	4497	15570
**Arrhythmia / Pro-arrhythmia, N(%)**						
Atrial fibrillation / flutter	19 (0.3)	60 (0.8)	75 (3.1)	60 (0.5)	94 (2.1)	154 (1.0)
Pro-AF condition*	45 (0.8)	105 (1.4)	92 (3.8)	107 (1.0)	135 (3.0)	242 (1.6)
Other arrhythmia / tachycardia	426 (7.3)	650 (8.9)	358 (14.7)	884 (8.0)	550 (12.2)	1434 (9.2)
Bradycardia / heart block	216 (3.7)	348 (4.8)	157 (6.4)	478 (4.3)	243 (5.4)	721 (4.6)
**Ischaemia, N(%)**						
Possible / probable	1305 (22.3)	2288 (31.4)	907 (37.2)	2782 (25.1)	1718 (38.2)	4500 (28.9)
Definite	97 (1.7)	137 (1.9)	73 (3.0)	193 (1.7)	114 (2.5)	307 (2.0)
**Left ventricular hypertrophy, N(%)**	364 (6.2)	835 (11.5)	382 (15.7)	937 (8.5)	644 (14.3)	1581 (10.2)
**Non-specific ECG changes, N(%)**	525 (9.0)	815 (11.2)	425 (17.4)	1110 (10.0)	655 (14.6)	1765 (11.3)
**Normal variant, N(%)**	3309 (56.7)	3355 (46.0)	777 (31.9)	5846 (52.8)	1595 (35.5)	7441 (47.8)

Percentages are calculated separately within columns.
ECG = electrocardiogram; AF = atrial fibrillation.
*Pro-AF condition = phenotype comprising ECG features that may increase the risk of development of atrial fibrillation.

Table S3. Prevalence of ECG phenotypes by 5 urban and 5 rural areas

	Urban						Rural						
	**Qingdao**	**Harbin**	**Haikou**	**Suzhou**	**Liuzhou**	**All**	**Sichuan**	**Gansu**	**Henan**	**Zhejiang**	**Hunan**	**All**	**All**
**Number of participants**	1651	2232	1402	2860	2795	10940	2797	2501	3109	2961	2931	14299	25239
**Arrhythmia / Pro-arrhythmia, N(%)**													
Atrial fibrillation / flutter	42 (2.5)	29 (1.3)	26 (1.9)	25 (0.9)	27 (1.0)	149 (1.4)	40 (1.4)	25 (1.0)	27 (0.9)	36 (1.2)	29 (1.0)	157 (1.1)	306 (1.2)
Pro-AF condition*	65 (3.9)	55 (2.5)	44 (3.1)	51 (1.8)	61 (2.2)	276 (2.5)	28 (1.0)	37 (1.5)	65 (2.1)	67 (2.3)	81 (2.8)	278 (1.9)	554 (2.2)
Other arrhythmia / tachycardia	106 (6.4)	198 (8.9)	109 (7.8)	235 (8.2)	227 (8.1)	875 (8.0)	278 (9.9)	217 (8.7)	274 (8.8)	339 (11.4)	305 (10.4)	1413 (9.9)	2288 (9.1)
Bradycardia / heart block	136 (8.2)	127 (5.7)	73 (5.2)	125 (4.4)	146 (5.2)	607 (5.5)	189 (6.8)	133 (5.3)	282 (9.1)	210 (7.1)	172 (5.9)	986 (6.9)	1593 (6.3)
**Ischaemia, N(%)**													
Possible / probable	536 (32.5)	478 (21.4)	360 (25.7)	729 (25.5)	816 (29.2)	2919 (26.7)	503 (18.0)	514 (20.6)	999 (32.1)	716 (24.2)	835 (28.5)	3567 (24.9)	6486 (25.7)
Definite	38 (2.3)	37 (1.7)	58 (4.1)	117 (4.1)	52 (1.9)	302 (2.8)	71 (2.5)	41 (1.6)	83 (2.7)	49 (1.7)	78 (2.7)	322 (2.3)	624 (2.5)
**Left ventricular hypertrophy, N(%)**	42 (2.5)	180 (8.1)	160 (11.4)	608 (21.3)	223 (8.0)	1213 (11.1)	553 (19.8)	307 (12.3)	344 (11.1)	463 (15.6)	540 (18.4)	2207 (15.4)	3420 (13.6)
**Non-specific ECG changes, N(%)**	238 (14.4)	350 (15.7)	262 (18.7)	467 (16.3)	469 (16.8)	1786 (16.3)	373 (13.3)	380 (15.2)	414 (13.3)	478 (16.1)	509 (17.4)	2154 (15.1)	3940 (15.6)
**Normal variant, N(%)**	768 (46.5)	1135 (50.9)	613 (43.7)	1172 (41.0)	1180 (42.2)	4868 (44.5)	1286 (46.0)	1277 (51.1)	1275 (41.0)	1332 (45.0)	1154 (39.4)	6324 (44.2)	11192 (44.3)

Percentages are calculated separately within columns.
ECG = electrocardiogram; AF = atrial fibrillation.
*Pro-AF condition = phenotype comprising ECG features that may increase the risk of development of atrial fibrillation.

Table S4. Prevalence of ECG phenotypes by age and CHA_2DS_2-VASc score without Suzhou area

	Age (years) at ECG measurement			CHA_2DS_2-VASc		
**ECG phenotypes**	**<55**	**55-69**	**70+**	**<2**	**2+**	**All**
**Number of participants**	8154	10293	3932	11287	11092	22379
**Arrhythmia / Pro-arrhythmia, N(%)**						
Atrial fibrillation / flutter	32 (0.4)	92 (0.9)	157 (4.0)	63 (0.6)	218 (2.0)	281 (1.3)
Pro-AF condition*	91 (1.1)	212 (2.1)	200 (5.1)	146 (1.3)	357 (3.2)	503 (2.2)
Other arrhythmia / tachycardia	566 (6.9)	896 (8.7)	591 (15.0)	856 (7.6)	1197 (10.8)	2053 (9.2)
Bradycardia / heart block	417 (5.1)	693 (6.7)	358 (9.1)	747 (6.6)	721 (6.5)	1468 (6.6)
**Ischaemia, N(%)**						
Possible / probable	1628 (20.0)	2837 (27.6)	1292 (32.9)	2055 (18.2)	3702 (33.4)	5757 (25.7)
Definite	132 (1.6)	240 (2.3)	135 (3.4)	206 (1.8)	301 (2.7)	507 (2.3)
**Left ventricular hypertrophy, N(%)**	711 (8.7)	1428 (13.9)	673 (17.1)	1218 (10.8)	1594 (14.4)	2812 (12.6)
**Non-specific ECG changes, N(%)**	962 (11.8)	1562 (15.2)	949 (24.1)	1634 (14.5)	1839 (16.6)	3473 (15.5)
**Normal variant, N(%)**	4388 (53.8)	4471 (43.4)	1161 (29.5)	5870 (52.0)	4150 (37.4)	10020 (44.8)

Percentages are calculated separately within columns.
ECG = electrocardiogram; AF = atrial fibrillation.
*Pro-AF condition = phenotype comprising ECG features that may increase the risk of development of atrial fibrillation.

Figure S1: Associations of ECG phenotype with heart failure

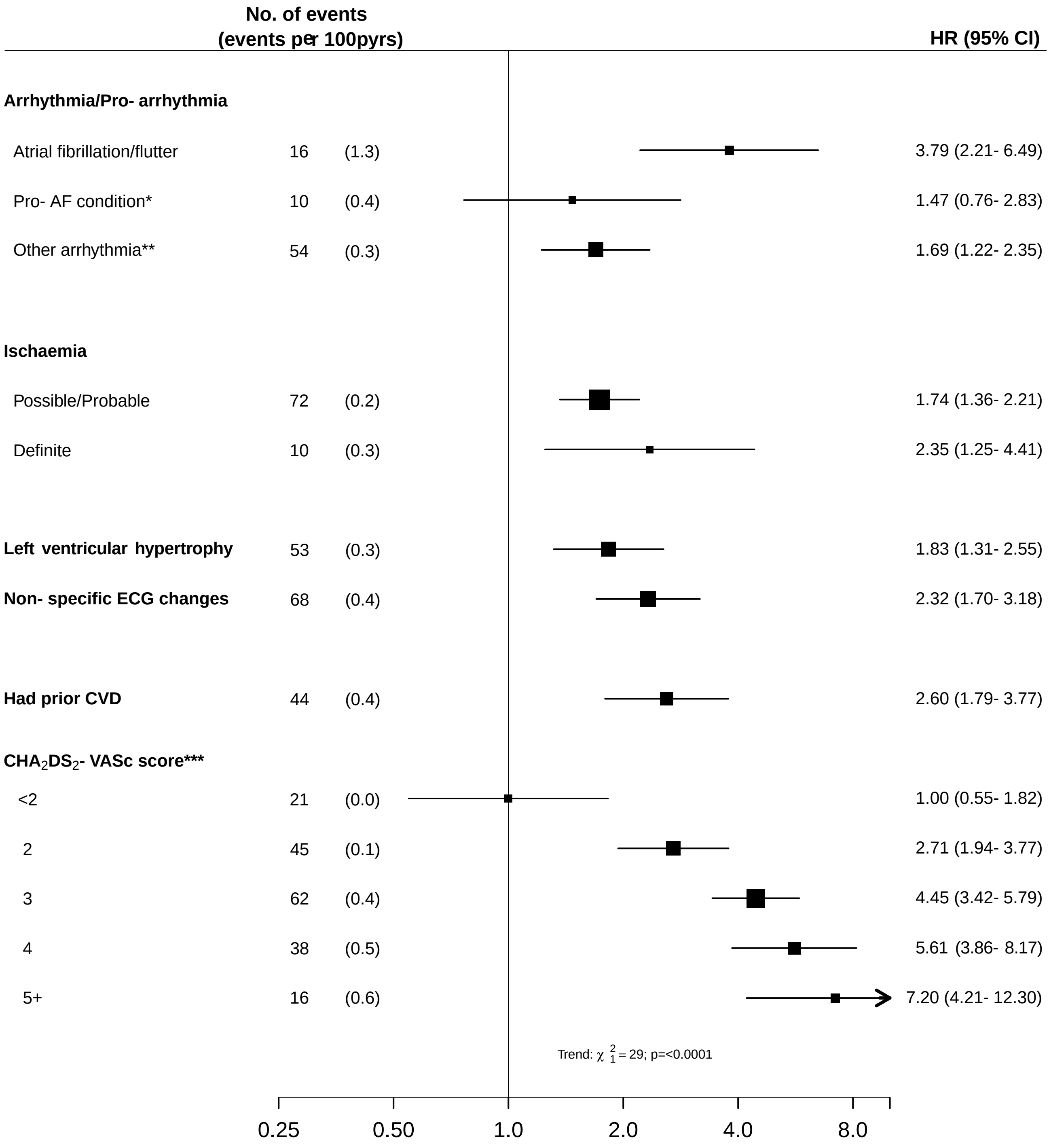

*Pro- AF condition = phenotype comprising ECG features that may increase the risk of development of atrial fibrillation.
**Includes other arrhythmia, tachycardia & brachycardia/heart block
***Reference (baseline) group is score < 2
ECG = electrocardiogram; AF = atrial fibrillation; CVD = cardiovascular disease

K:/kadoorie/Staff_Folders/JimH/2023- 09- 07/figures/eFig1.R 20SEP2023 13:26

Figure S2: Association of three ECG phenotypes with stroke, CVD, heart failure and IHD, with and without Suzhou area

	No. of events (events per 100pyrs)	HR (95% CI)
**Stroke**		
Left ventricular hypertrophy	320 (2.1)	1.26 (1.11- 1.42)
- excluding Suzhou	288 (2.4)	1.25 (1.09- 1.42)
Non- specific ECG changes	342 (2.0)	1.06 (0.94- 1.20)
- excluding Suzhou	326 (2.2)	1.08 (0.96- 1.23)
Ischaemia (All)	635 (2.1)	1.29 (1.17- 1.43)
- excluding Suzhou	595 (2.2)	1.30 (1.17- 1.44)
**Cardiovascular disease**		
Left ventricular hypertrophy	608 (4.5)	1.20 (1.10- 1.32)
- excluding Suzhou	556 (5.2)	1.20 (1.09- 1.32)
Non- specific ECG changes	712 (4.8)	1.20 (1.11- 1.31)
- excluding Suzhou	679 (5.4)	1.23 (1.12- 1.34)
Ischaemia (All)	1246 (4.7)	1.34 (1.25- 1.44)
- excluding Suzhou	1164 (5.2)	1.33 (1.23- 1.43)
**Heart failure**		
Left ventricular hypertrophy	53 (0.3)	1.83 (1.31- 2.55)
- excluding Suzhou	50 (0.4)	1.94 (1.38- 2.73)
Non- specific ECG changes	68 (0.4)	2.32 (1.70- 3.18)
- excluding Suzhou	60 (0.4)	2.24 (1.62- 3.10)
Ischaemia (All)	82 (0.2)	1.79 (1.33- 2.42)
- excluding Suzhou	75 (0.3)	1.74 (1.28- 2.38)
**Ischaemic heart disease**		
Left ventricular hypertrophy	244 (1.6)	1.15 (0.99- 1.32)
- excluding Suzhou	233 (1.9)	1.14 (0.99- 1.32)
Non- specific ECG changes	319 (1.9)	1.22 (1.07- 1.38)
- excluding Suzhou	311 (2.1)	1.22 (1.07- 1.39)
Ischaemia (All)	574 (1.9)	1.42 (1.28- 1.58)
- excluding Suzhou	551 (2.1)	1.40 (1.25- 1.56)

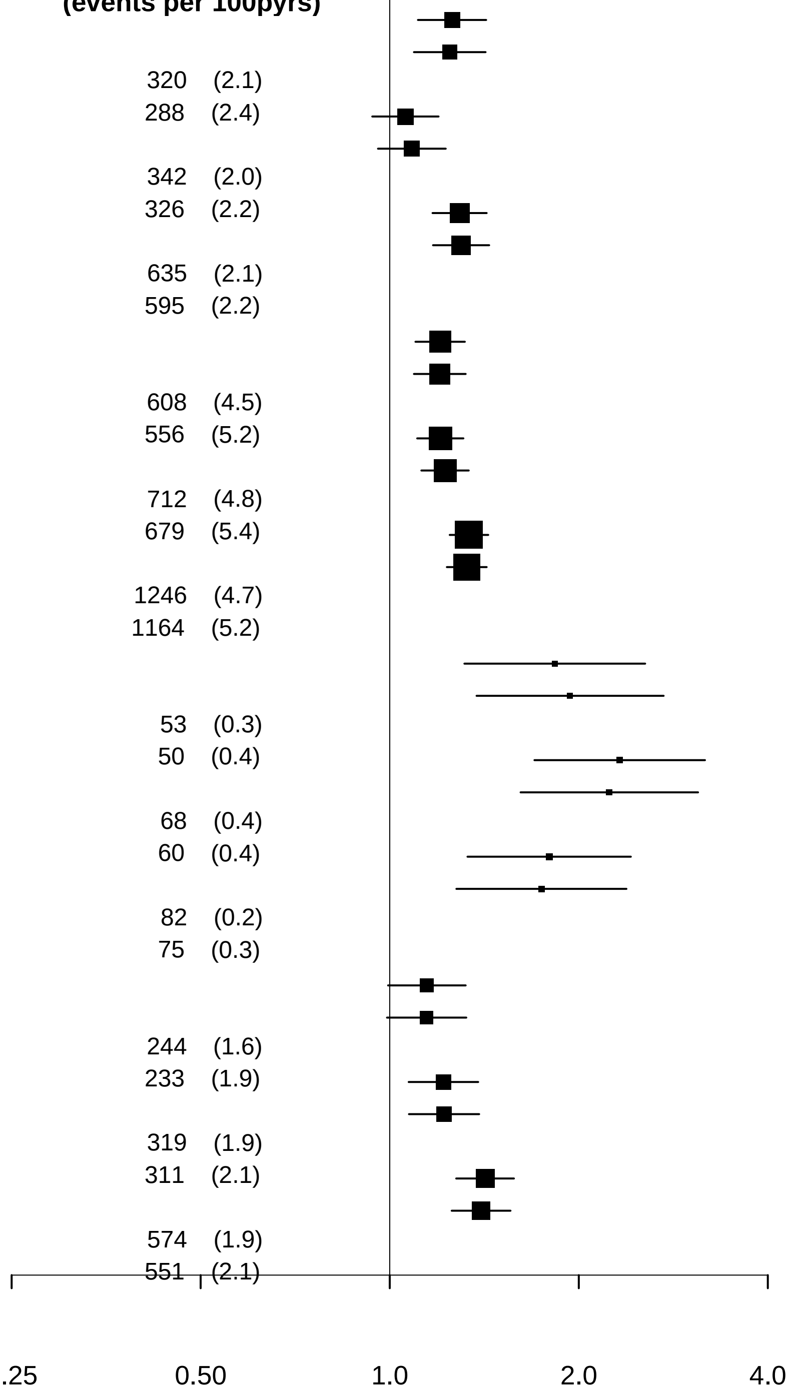

ECG = electrocardiogram; CVD = cardiovascular disease; IHD = ischaemic heart disease

K:/kadoorie/Staff_Folders/JimH/2023- 09- 07/figures/eFig2_excl_rc36.R 20SEP2023 15:02

Figure S3: Cumulative incidence of CVD for AF phenotype, only by rural vs urban

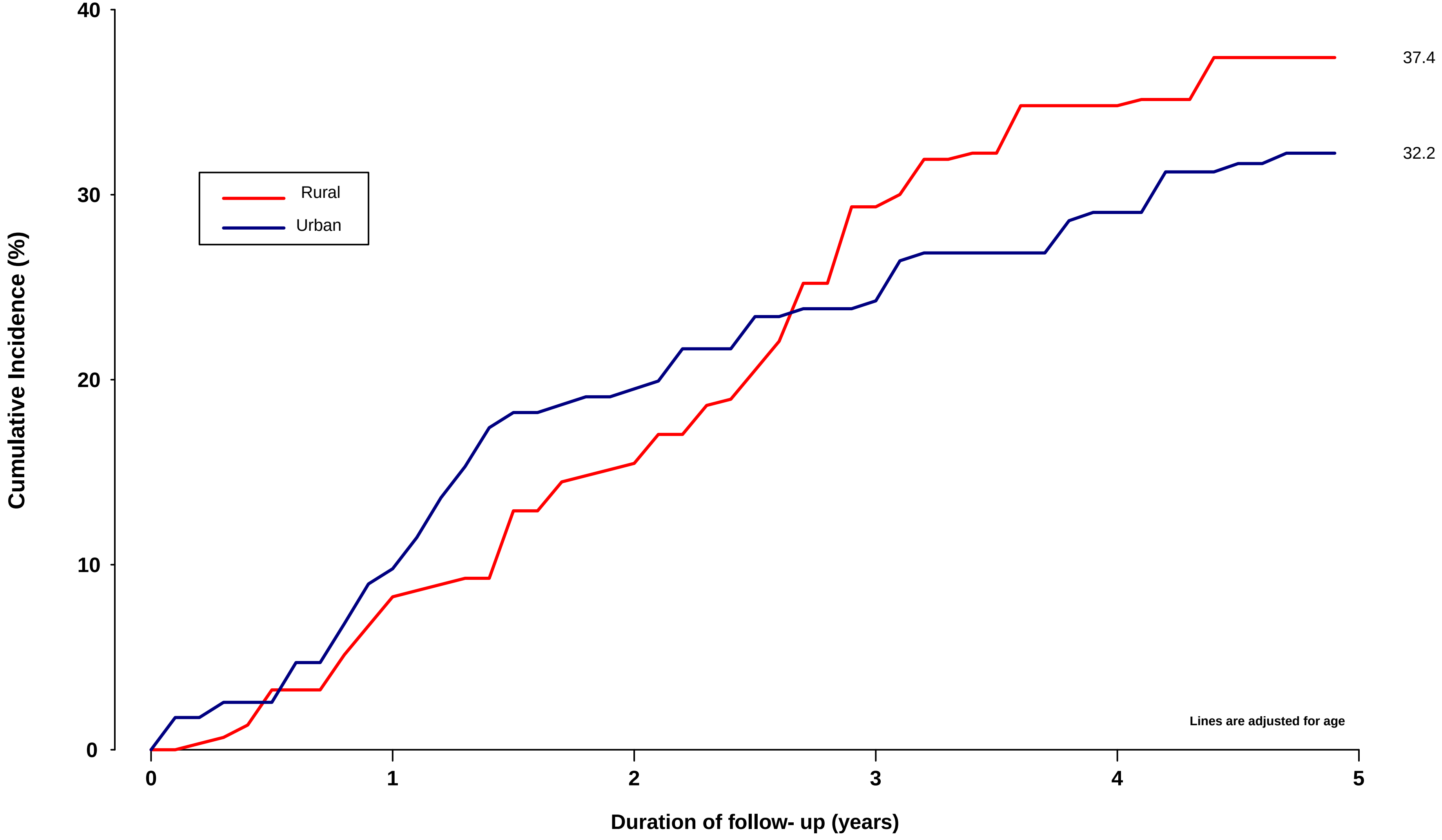

Number at risk						
Rural	82	72	59	55	48	25
Urban	92	81	72	59	51	20

CVD = cardiovascular disease; AF = atrial fibrillation

Figure S4: Cumulative incidence of CVD by sex

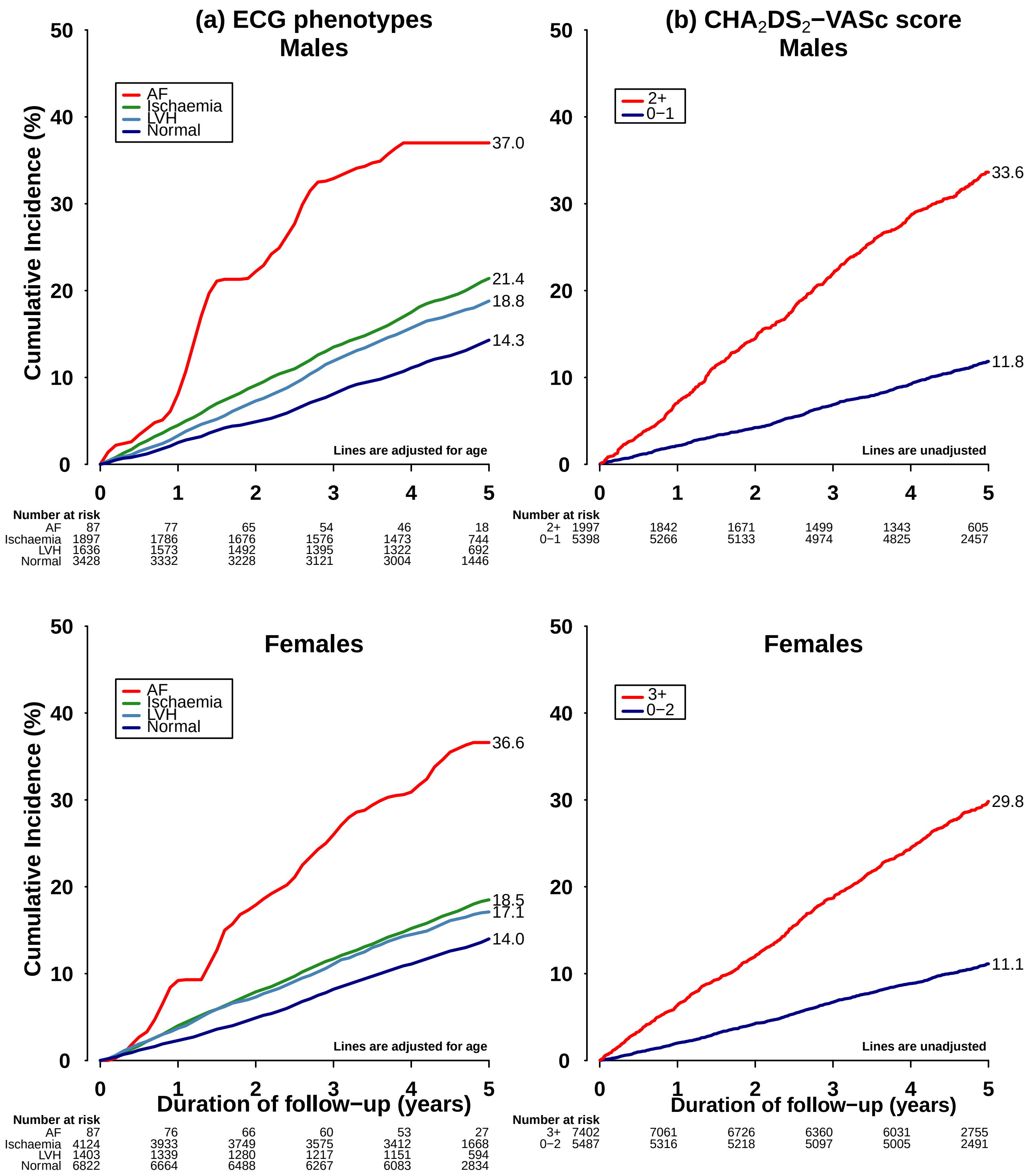

CVD = cardiovascular disease; ECG = electrocardiogram; AF = atrial fibrillation; LVH = left ventricular hypertrophy

Figure S5: Cumulative incidence of CVD by status of self−reported prior CVD

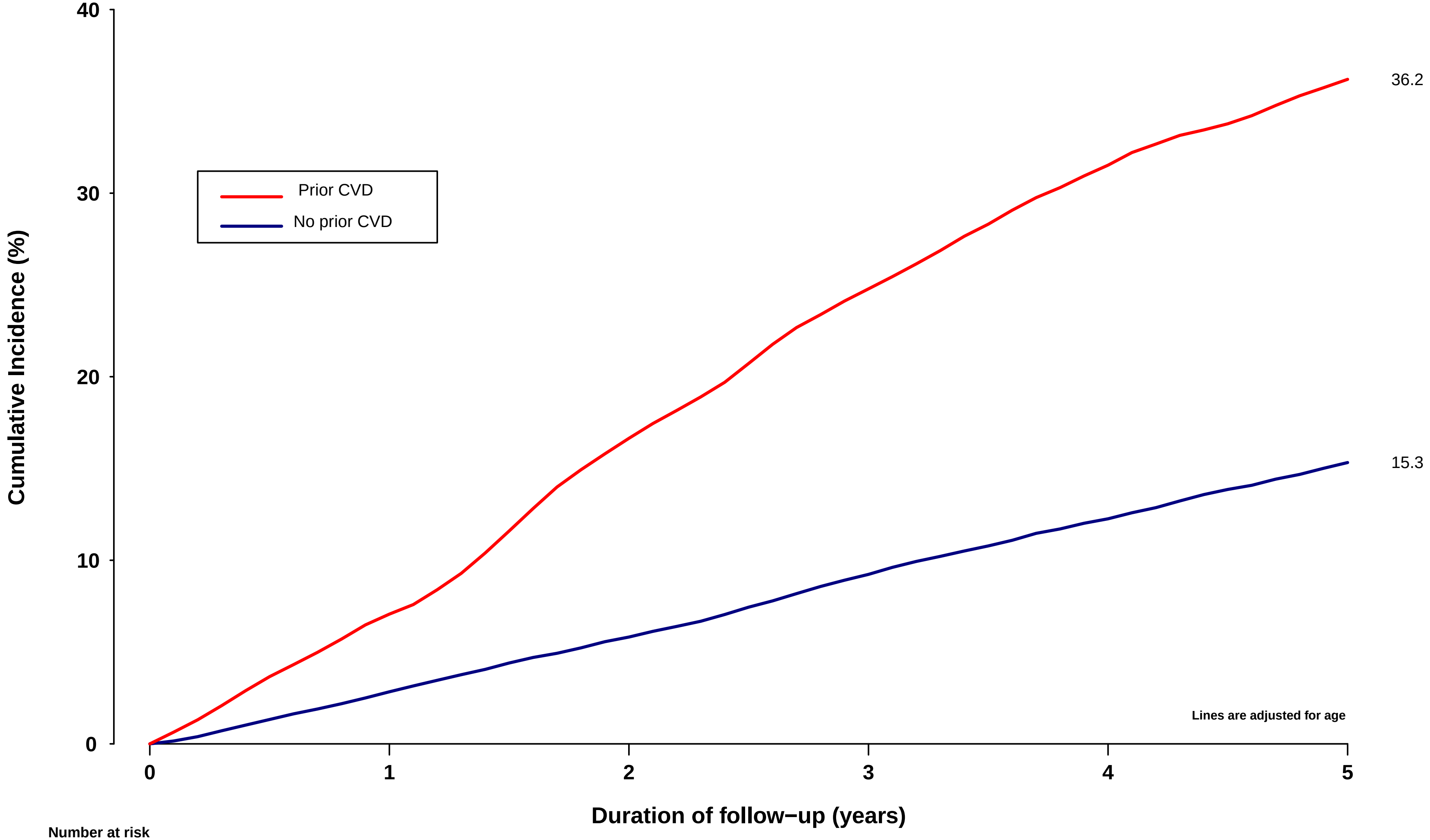

Number at risk

	0	1	2	3	4	5
Prior CVD	1099	997	885	766	679	295
No prior CVD	21089	20402	19693	18887	18187	8803

CVD = cardiovascular disease

Figure S6: Cumulative incidence of CVD with Suzhou area excluded

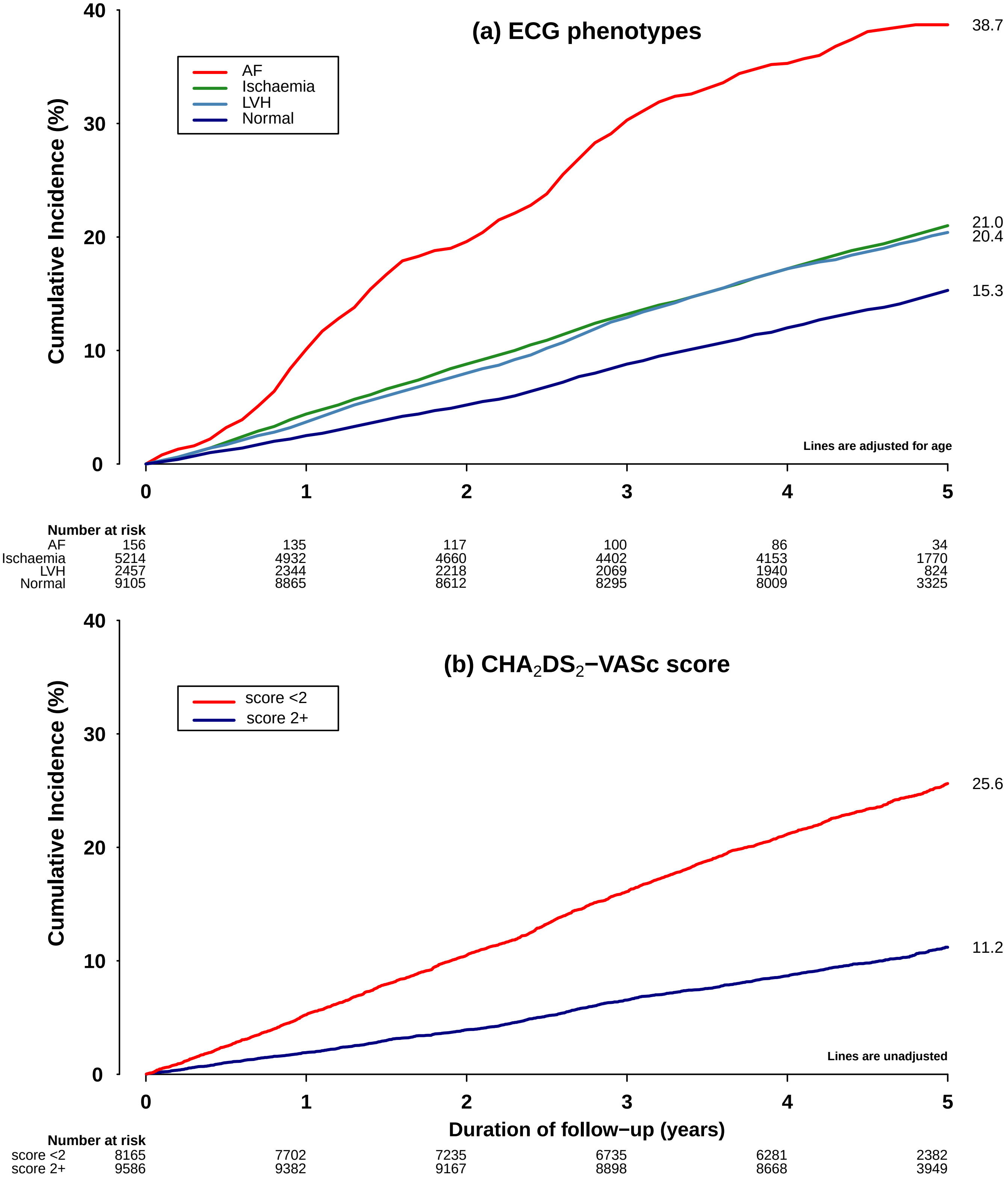

CVD = cardiovascular disease; ECG = electrocardiogram; AF = atrial fibrillation; LVH = left ventricular hypertrophy
